# Supplementary material for: Network propagation of rare variants in Alzheimer’s disease reveals tissue-specific hub genes and communities
Source: PLoS Comput Biol. 2021 Jan 7;17(1):e1008517. doi: 10.1371/journal.pcbi.1008517 (PMC7817020; doi:10.1371/journal.pcbi.1008517)
Supplement: S1 Text — (DOCX) [file pcbi.1008517.s001.docx]

# Supplementary Methods

# Genetic data processing

*ADNI WGS data preprocessing*

WGS was obtained from blood genomic DNA samples and processed by ADNI, as described elsewhere [1]. Briefly, sequencing was performed using the Illumina HiSeq2000 system through paired-end read chemistry and read lengths of 100 base pairs. Reads were aligned to the reference human genome (NCBI build 37.72) using the BWA tool [2] and used for multi-sample variant calling with the GATK HaplotypeCaller [3].

*ADSP WES data preprocessing*

ADSP WES data were retrieved through dbGaP (accession ID: phs000572.v7.p4), which include QC'ed SNV genotypes concordant between the Atlas (Baylor's) and GATK (Broad's) calling pipelines. Briefly, 10,913 subjects underwent WES at three different sequencing centers (the Human Genome Sequencing Center at Baylor College of Medicine, Broad Institute, and Genome Institute at Washington University), capturing the exome target region using the Illumina Rapid Capture Exome or the Nimblegen's VCRome v2.1 exome kits and paired-end sequencing them on Illumina HiSeq 2000 platform. Detailed information about sequencing and QC pipeline is available from <https://www.niagads.org/adsp/content/sequencing-pipelines>. Whole-genome sequencing (WGS) data, available from ADSP was not considered since the majority of the subjects available were of Caribbean-Hispanic ancestry.

We identified ADSP subjects who were sequenced as part of ADNI and removed them from the sample. To identify overlapping samples, we performed the following steps: we selected from the ADNI WGS data the set of exonic SNVs in common with ADSP WES, then merged the two datasets using PLINK v1.9 [4]. On the merged dataset, we performed basic quality control (minor allele frequency [MAF] <0.05, SNV missingness rate >0.1, Hardy-Weinberg equilibrium p<5e-7), followed by linkage disequilibrium-based pruning (PLINK parameters: --indep-pairwise 500 50 0.2). On this dataset of independent exonic SNVs, we computed identity-by-descent for all pairs of samples, and identified as duplicates all ADSP subjects exhibiting PI_HAT > 0.95 (PLINK parameters: --genome --min 0.95).

# Tissue-specific gene interaction networks

As a substrate for network propagation, we leveraged tissue-specific weighted gene interaction networks from Greene et al. [5]. In these networks, each node represents a gene, each edge a functional relationship, and an edge between two genes is probabilistically weighted based on experimental evidence connecting both genes.

Curation of these networks involved the integration of evidence from 987 genome-scale data sets encompassing approximately 38,000 conditions from an estimated 14,000 publications including both expression and interaction measurements in 144 tissues and cell lineages, with each data set weighted in a process specific manner. Tissue-specific gene interaction networks are freely available for download from .http://hb.flatironinstitute.org/download.

# Network propagation - synthetic data

We conducted a set of experiments with simulated gene mutation profiles to examine the behaviour of our network propagation implementation, as well as to determine the optimal values for some of the parameters listed in the previous section.

Simulation and investigation of the parameter space was carried out jointly in three nested levels.

***Level 1*:** the original, weighted network is read in; the network is then binarised retaining a certain percentage P of top edges. We vary $P \in\left\{ 0.5;1;5;10;25 \right\}.$ For the sake of computational speed, we do not focus on the entire hippocampus gene network; instead, from the binarised network we extract a subgraph of radius 2 centered on a randomly selected hub gene (in this instance, *CALML3*), using the python package *networkx* version 2.2 [6]. This subgraph extraction is performed only for simulation purposes, whereas the full hippocampus network is used for application to real world data. The number M of nodes in this subgraph is retained for use in the next level.

***Level 2*:** synthetic, binary mutation profiles for S = 2,000 subjects (equally divided into AD cases and controls) and M genes are generated by assigning the “mutated” status to proportions $f_{HC}$ and $f_{AD}$ of controls and cases randomly for each gene. We vary the

gene-level mutation frequencies in the following ranges:

- mutation frequency in controls $f_{HC}\in\left\{ 0;0.1;0.5 \right\}\%$
- mutation frequency in AD cases $f_{AD}\left\{ 0.1;0.5;1 \right\}\%$

Additionally, we simulate three different scenarios for the propagation of mutation signals:

1. only the first neighbours of the hub gene are mutated;
2. only the second neighbours of the hub gene are mutated;
3. both first and second neighbours are mutated (realistic scenario).

This results in 27 synthetic mutation profiles generated. This is repeated also allowing the hub gene to be mutated.

***Level 3:*** for each of the 27 previously generated synthetic mutation profiles, network propagation is run by varying:

- the diffusion length $\alpha\in\left\{ 0;0.25;0.5;0.75;0.9 \right\};$
- whether quantile normalisation is applied or not to the final smooth profile. Quantile normalisation was used in Hofree et al. [7] for clustering purposes; however, we sought to assess its impact for the purpose of association testing.

Lastly, after network propagation was run with the selected set of parameters on the simulated data, the smoothed score for the hub gene was tested for difference between cases and controls with a Wilcoxon rank-sum test. Statistical significance was established at p < 0.05.

# Differential gene expression analysis

RNA sequencing and processing at the Mayo Clinic Brain Bank was described in detail elsewhere ([8] and [https://www.synapse.org/#!Synapse:syn3163039](https://www.synapse.org/#!Synapse%3Asyn3163039) ). Each sample was assigned one of the following pathological diagnoses: Alzheimer’s disease (AD, N=84), progressive supranuclear palsy (PSP, N=84), pathologic aging (PA, N=30), and control (HC, N=80). We only focused on differential expression analysis for HC vs AD (total N = 156 after sample QC). Normalized read counts were assessed for differential expression between diagnosis groups, using multi-variable linear regression adjusting for key covariates. Two models were run for each comparison called “Simple” (syn6090804) and “Comprehensive” (syn6090803). The simple model includes as covariates: age at death, sex, RNA integrity number (RIN), Source and FLOWCELL (syn3817650). The comprehensive model includes the same covariates plus normalized counts for 5 genes as surrogate variables for relevant cell types as follows: *CD68* (Microglia), *CD34* (Endothelial cells), *OLIG2* (Oligodendroglia), *GFAP* (Astrocytes) and *ENO2* (Neurons).

RNA sequencing and processing at the Mount Sinai Brain Bank was described in detail elsewhere ([9] and [https://www.synapse.org/#!Synapse:syn3157743](https://www.synapse.org/#!Synapse%3Asyn3157743)). Gene expression levels were normalised by regressing out the effect of sex, race, age at death, post-mortem interval, RIN, exonic rate, rRNA rate and batch. The accession code for normalised expression levels in Brodmann area 36 is syn16795937. We also downloaded RNA-seq covariates (syn6100548) and post-mortem clinical assessments (syn6101474). Each sample was assigned a neuropathology category according to the Consortium to Establish a Registry for Alzheimer's Disease (CERAD) protocol (1=normal, 2=definite AD, 3=probable AD, 4=possible AD) [10].

**References**

1. Saykin AJ, et al. (2015) Genetic studies of quantitative MCI and AD phenotypes in ADNI: Progress, opportunities, and plans. *Alzheimer’s Dement* 11(7). doi:10.1016/j.jalz.2015.05.009.

2. Li H, Durbin R (2009) Fast and accurate short read alignment with Burrows- Wheeler transform. *Bioinformatics*. doi:10.1093/bioinformatics/btp324.

3. Depristo MA, et al. (2011) A framework for variation discovery and genotyping using next-generation DNA sequencing data. *Nat Genet*. doi:10.1038/ng.806.

4. Chang CC, et al. (2015) Second-generation PLINK: rising to the challenge of larger and richer datasets. *Gigascience* 4(1):7.

5. Greene CS, et al. (2015) Understanding multicellular function and disease with human tissue-specific networks. *Nat Genet* 47(6):569–76.

6. Hagberg AA, Schult DA, Swart PJ (2008) Exploring network structure, dynamics, and function using NetworkX. *Proceedings of the 7th Python in Science Conference (SciPy)* doi:10.1016/j.jelectrocard.2010.09.003.

7. Hofree M, Shen JP, Carter H, Gross A, Ideker T (2013) Network-based stratification of tumor mutations. *Nat Methods* 10(11):1108–1115.

8. Allen M, et al. (2016) Human whole genome genotype and transcriptome data for Alzheimer’s and other neurodegenerative diseases. *Sci Data* 3:160089.

9. Wang M, et al. (2018) The Mount Sinai cohort of large-scale genomic, transcriptomic and proteomic data in Alzheimer’s disease. *Sci Data* 5:180185.

10. Mirra SS, et al. (1991) The Consortium to Establish a Registry for Alzheimer’s Disease (CERAD). Part II. Standardization of the neuropathologic assessment of Alzheimer’s disease. *Neurology* 41(4):479–86.
