# Supplementary material for: Network propagation of rare variants in Alzheimer’s disease reveals tissue-specific hub genes and communities
Source: PLoS Comput Biol. 2021 Jan 7;17(1):e1008517. doi: 10.1371/journal.pcbi.1008517 (PMC7817020; doi:10.1371/journal.pcbi.1008517)
Supplement: S1 Table — If a gene did not have any SNVs mapped to it, and therefore was not included in network propagation, this resulted in missing selection probabilities (NA values). (DOCX) [file pcbi.1008517.s003.docx]

**Supporting Information**

**Table S1** – Selection probabilities (from the application of NETPAGE to ADSP) for the 21 genes reported in the recent GWAS by Kunkle et al. [24]**.** If a gene did not have any SNVs mapped to it, and therefore was not included in network propagation, this resulted in missing selection probabilities (NA values).

| **Gene** | **Selection probability** | **Gene** | **Selection probability** |
| --- | --- | --- | --- |
| *CR1* | 0.00 | *FERMT2* | 0.00 |
| *BIN1* | 0.00 | *SLC24A4* | 0.00 |
| *INPP5D* | NA | *ABCA7* | 0.00 |
| *HLA-DRB1* | 0.00 | *APOE* | 0.00 |
| *TREM2* | 0.99 | *CASS4* | 0.00 |
| *CD2AP* | 0.00 | *ECHDC3* | 0.00 |
| *NYAP1* | 0.00 | *ACE* | 0.00 |
| *EPHA1* | 0.00 | *NDUFAF6* | 0.00 |
| *PTK2B* | 0.00 | *ADAM10* | 0.02 |
| *CLU* | 0.00 | *IQCK* | 0.00 |
| *SPI1* | 0.00 | *MIR142* | NA |
| *MS4A2* | 0.00 | *ADAMTS1* | 0.00 |
| *PICALM* | 0.18 | *OARD1* | 0.00 |
| *SORL1* | 0.00 | *WWOX* | 0.00 |
