# Supplementary material for: Network propagation of rare variants in Alzheimer’s disease reveals tissue-specific hub genes and communities
Source: PLoS Comput Biol. 2021 Jan 7;17(1):e1008517. doi: 10.1371/journal.pcbi.1008517 (PMC7817020; doi:10.1371/journal.pcbi.1008517)
Supplement: S2 Table — The set of interest was formed by including the first interaction neighbours of PFAS. Additionally, the first two rows in the table report gene-based p-values for PFAS from S7 Fig. (DOCX) [file pcbi.1008517.s004.docx]

**Supporting Information**

**Table S2 -** Results of set-based SKAT test for association of rare, exonic, deleterious variants with case-control status in ADNI. The set of interest was formed by including the first interaction neighbours of PFAS. Additionally, the first two rows in the table report gene-based p-values for PFAS from S7 Fig.

| **Test** | **PFAS p-value** |
| --- | --- |
| Omnibus (SKAT-O), gene-based | 0.39 |
| Smoothed logistic, gene-based | 0.001 |
| Burden, set-based | 0.82 |
| Variance-component, set-based | 0.43 |
| Omnibus (SKAT-O), set-based | 0.66 |
