# Supplementary material for: Network propagation of rare variants in Alzheimer’s disease reveals tissue-specific hub genes and communities
Source: PLoS Comput Biol. 2021 Jan 7;17(1):e1008517. doi: 10.1371/journal.pcbi.1008517 (PMC7817020; doi:10.1371/journal.pcbi.1008517)
Supplement: S3 Table — (DOCX) [file pcbi.1008517.s005.docx]

**Supporting Information**

**Table S3** – Degree of the 30 genes selected in ADNI and ADSP in the hippocampus functional network.

| Gene | Degree | Gene | Degree |
| --- | --- | --- | --- |
| *ABR* | 285 | *KCNMA1* | 297 |
| *ADRM1* | 299 | *KLC1* | 403 |
| *APPBP2* | 182 | *MAPK11* | 76 |
| *ARL1* | 361 | *MAPRE1* | 227 |
| *ATXN10* | 105 | *MAPRE3* | 144 |
| *CAMK2B* | 219 | *PFAS* | 41 |
| *CAPNS1* | 207 | *MOB4* | 270 |
| *COPS5* | 389 | *MRPL17* | 80 |
| *CSNK1A1* | 278 | *PPP1CC* | 298 |
| *CUL5* | 159 | *RAB1A* | 382 |
| *DCTN6* | 135 | *SHOC2* | 365 |
| *DSTN* | 163 | *TMEM147* | 339 |
| *EFNB3* | 207 | *TREM2* | 0 |
| *GNB1* | 412 | *UBL3* | 154 |
| *HIC2* | 153 | *ZNF207* | 190 |
