# Supplementary material for: Network propagation of rare variants in Alzheimer’s disease reveals tissue-specific hub genes and communities
Source: PLoS Comput Biol. 2021 Jan 7;17(1):e1008517. doi: 10.1371/journal.pcbi.1008517 (PMC7817020; doi:10.1371/journal.pcbi.1008517)
Supplement: S4 Table — The gene set tested for overrepresentation included PFAS and its first and second interaction neighbours in the thresholded and binarised hippocampus functional network. Hits = number of observed genes overlapping with the curated gene set of interest; expected = number of genes expected to overlap with the curated gene set of interest by chance; OR = odds ratio from the Fisher’s test; P = p-value of the Fisher’s test; Pcorr = p-value corrected with the Benjamini-Hochberg procedure. P-values reported in bold are significant at FDR 5%. (DOCX) [file pcbi.1008517.s006.docx]

**Supporting Information**

**Table S4 -** Results of gene set enrichment analysis for eight AD-related gene sets. The gene set tested for overrepresentation included PFAS and its first and second interaction neighbours in the thresholded and binarised hippocampus functional network. Hits = number of observed genes overlapping with the curated gene set of interest; expected = number of genes expected to overlap with the curated gene set of interest by chance; OR = odds ratio from the Fisher’s test; P = p-value of the Fisher’s test; P_corr_ = p-value corrected with the Benjamini-Hochberg procedure. P-values reported in bold are significant at FDR 5%.

| **Gene set** | **Hits** | **Expected** | **OR** | **P** | **P_corr_ (FDR)** |
| --- | --- | --- | --- | --- | --- |
| Alzheimer’s disease (KEGG) | 22 | 9.68 | 2.70 | 0.00016 | **0.00145** |
| Blalock - upregulated in incipient AD (CGP) | 242 | 127.47 | 2.35 | 6.18E-25 | **4.18E-23** |
| Blalock - downregulated in AD (CGP) | 214 | 75.25 | 4.12 | 9.39E-50 | **2.33E-47** |
| Blalock - upregulated in AD (CGP) | 227 | 178.01 | 1.38 | 0.00003 | **0.00077** |
| Blalock - downregulated in incipient AD (CGP) | 33 | 10.87 | 4.09 | 2.35E-9 | **4.25E-8** |
| Ray - Alzheimer’s disease (CGP) | 0 | 0.86 | 0 | 1 | 1 |
| Wu - upregulated in AD (CGP) | 0 | 0.86 | 0 | 1 | 1 |
| Wu - downregulated in AD (CGP) | 3 | 1.07 | 3.55 | 0.08392 | 0.32 |
