# Supplementary material for: Network propagation of rare variants in Alzheimer’s disease reveals tissue-specific hub genes and communities
Source: PLoS Comput Biol. 2021 Jan 7;17(1):e1008517. doi: 10.1371/journal.pcbi.1008517 (PMC7817020; doi:10.1371/journal.pcbi.1008517)
Supplement: S5 Table — The + or–sign in brackets next to the p-value represents the direction of effect detected. Pairwise comparisons were performed for normalised expression levels against CERAD diagnosis using the nonparametric Dunn’s test. Values in boldface are the ones that remained significant after a two-fold multiple testing correction (Benjamini-Hochberg for the multiple pairwise comparisons for a given gene, Bonferroni for the number of genes tested). In total, 16 genes were seen to be significantly dysregulated in at least one comparison. (DOCX) [file pcbi.1008517.s007.docx]

**Supporting Information**

**Table S5** - Differential expression analysis uncorrected, two-tailed p-values on the MSBB RNA-seq dataset for the 30 genes selected from ADNI and ADSP. The + or – sign in brackets next to the p-value represents the direction of effect detected. Pairwise comparisons were performed for normalised expression levels against CERAD diagnosis using the nonparametric Dunn’s test. Values in boldface are the ones that remained significant after a two-fold multiple testing correction (Benjamini-Hochberg for the multiple pairwise comparisons for a given gene, Bonferroni for the number of genes tested). In total, 16 genes were seen to be significantly dysregulated in at least one comparison.

| **Gene** | **Normal vs definite AD** | **Normal vs probable AD** | **Normal vs possible AD** | **Definite vs probable AD** | **Definite vs possible AD** | **Probable vs possible AD** |
| --- | --- | --- | --- | --- | --- | --- |
| *ABR* | 0.08 (+) | 0.43 (+) | 0.19 (+) | 0.55 (-) | 0.93 (+) | 0.60 (+) |
| *ADRM1* | 0.06 (+) | 0.07 (+) | 0.53 (-) | 0.70 (+) | 0.04 (-) | 0.04 (-) |
| *APPBP2* | 0.02 (+) | 0.20 (+) | 0.31 (-) | 0.62 (-) | 0.007 (-) | 0.06 (-) |
| *ARL1* | **9.60E-05 (+)** | 0.06 (+) | 0.52 (-) | 0.21 (-) | **4E-4 (-)** | 0.04 (-) |
| *ATXN10* | **2.80E-05 (+)** | 0.04 (+) | 0.35 (-) | 0.21 (-) | **6.43E-05 (-)** | 0.01 (-) |
| *CAMK2B* | **1.68E-4 (+)** | 0.15 (+) | 0.27 (+) | 0.12 (-) | 0.10 (-) | 0.84 (-) |
| *CAPNS1* | **2.54E-4 (+)** | 0.07 (+) | 0.62 (-) | 0.27 (-) | 1.55E-3 (-) | 0.06 (-) |
| *COPS5* | 7E-3 (+) | 0.20 (+) | 0.09 (-) | 0.39 (-) | **2.54E-4 (-)** | 0.01 (-) |
| *CSNK1A 1* | 0.22 (+) | 0.17 (+) | 9.41E-3 (-) | 0.66 (+) | 3.73E-4 (-) | 6.74E-4 (-) |
| *CUL5* | 0.05 (+) | 0.30 (+) | 0.14 (-) | 0.61 (-) | 0.004 (-) | 0.04 (-) |
| *DCTN6* | 0.002 (+) | 0.11 (+) | 0.47 (-) | 0.43 (-) | 0.004 (-) | 0.06 (-) |
| *DSTN* | 4.2E-4 (+) | 0.08 (+) | 0.35 (-) | 0.29 (-) | **4.21E-4 (-)** | 0.03 (-) |

| *EFNB3* | **9.13E-05 (+)** | 0.64 (+) | 0.90 (-) | 0.007 (-) | 0.003 (-) | 0.63 (-) |
| --- | --- | --- | --- | --- | --- | --- |
| *GNB1* | **2.27E-06 (+)** | 0.01 (+) | 0.51 (+) | 0.23 (-) | 0.005 (-) | 0.14 (-) |
| *HIC2* | 0.42 (-) | 0.15 (-) | 0.33 (+) | 0.39 (-) | 0.11 (+) | 0.04 (+) |
| *KCNMA1* | **7.86E-05 (+)** | 7.6E-4 (+) | 0.24 (+) | 0.75 (+) | 0.09 (-) | 0.09 (-) |
| *KLC1* | **1.6E-4 (+)** | 0.74 (+) | 0.83 (-) | 0.007 (-) | 0.003 (-) | 0.65 (-) |
| *MAPK11* | 9.02E-4 (+) | 0.29 (+) | 0.10 (+) | 0.11 (-) | 0.45 (-) | 0.55 (+) |
| *MAPRE1* | **3.48E-05 (-)** | 0.42 (-) | 0.56 (-) | 0.01 (+) | 0.01 (+) | 0.89 (+) |
| *MAPRE3* | **2.25E-05 (+)** | 0.11 (+) | 0.46 (+) | 0.07 (-) | 0.02 (-) | 0.53 (-) |
| *MOB4* | **1.05E-4 (+)** | 0.01 (+) | 0.36 (-) | 0.50 (-) | **1.72E-4 (-)** | 0.006 (-) |
| *MRPL17* | 8.42E-4 (+) | 0.70 (+) | 0.03 (-) | 0.02 (-) | **2.82E-06 (-)** | 0.02 (-) |
| *PFAS* | 0.04 (+) | 0.71 (-) | 0.43 (+) | 0.04 (-) | 0.51 (-) | 0.32 (+) |
| *PPP1CC* | 0.05 (-) | 0.56 (-) | 0.67 (-) | 0.34 (+) | 0.33 (+) | 0.92 (+) |
| *RAB1A* | 0.002 (+) | 0.10 (+) | 0.17 (-) | 0.42 (-) | **2.71E-4 (-)** | 0.01 (-) |
| *SHOC2* | 0.001 (+) | 0.04 (+) | 0.52 (-) | 0.69 (-) | 0.003 (-) | 0.02 (-) |
| *TMEM14 7* | 0.06 (+) | 0.61 (+) | 0.15 (-) | 0.34 (-) | 0.005 (-) | 0.09 (-) |
| *TREM2* | **4.52E-06 (-)** | 0.26 (-) | 0.05 (-) | 0.01 (+) | 0.16 (+) | 0.43 (-) |
| *UBL3* | 0.79 (+) | 0.75 (+) | 0.34 (-) | 0.91 (+) | 0.24 (-) | 0.27 (-) |
| *ZNF207* | 0.05 (-) | 0.67 (-) | 0.13 (-) | 0.27 (+) | 0.86 (-) | 0.31 (-) |
