# Supplementary material for: Network propagation of rare variants in Alzheimer’s disease reveals tissue-specific hub genes and communities
Source: PLoS Comput Biol. 2021 Jan 7;17(1):e1008517. doi: 10.1371/journal.pcbi.1008517 (PMC7817020; doi:10.1371/journal.pcbi.1008517)

**Supporting Information**

**Figure S1** - A selection of simulation results. The x and y axis represent the mutation frequencies in controls and cases respectively. The faceting allows to visualise the effect of other parameters (diffusion length, percentage of edges retained, quantile normalisation). The colour-coding indicates the statistical significance of the difference in the hub gene smoothed score between controls and cases, in units of -log10 p-value. We investigated three mutation scenarios: scenario 1, only first neighbours mutated; scenario 2, only second neighbours mutated; scenario 3, both first and second neighbours mutated; the target gene is always unmutated. Cells marked with a black cross indicate parameter combinations where the smooth score of the hub gene was not significantly different between cases and controls. (A) Effect of quantile normalisation after network propagation with top 1% edges retained (top row, scenario 1 on the left, scenario 3 on the right), and with top 25% edges retained (bottom row, mutation scenarios as in top row). (B) Joint effect of percentage of edges retained and diffusion length for the three mutation scenarios considered.


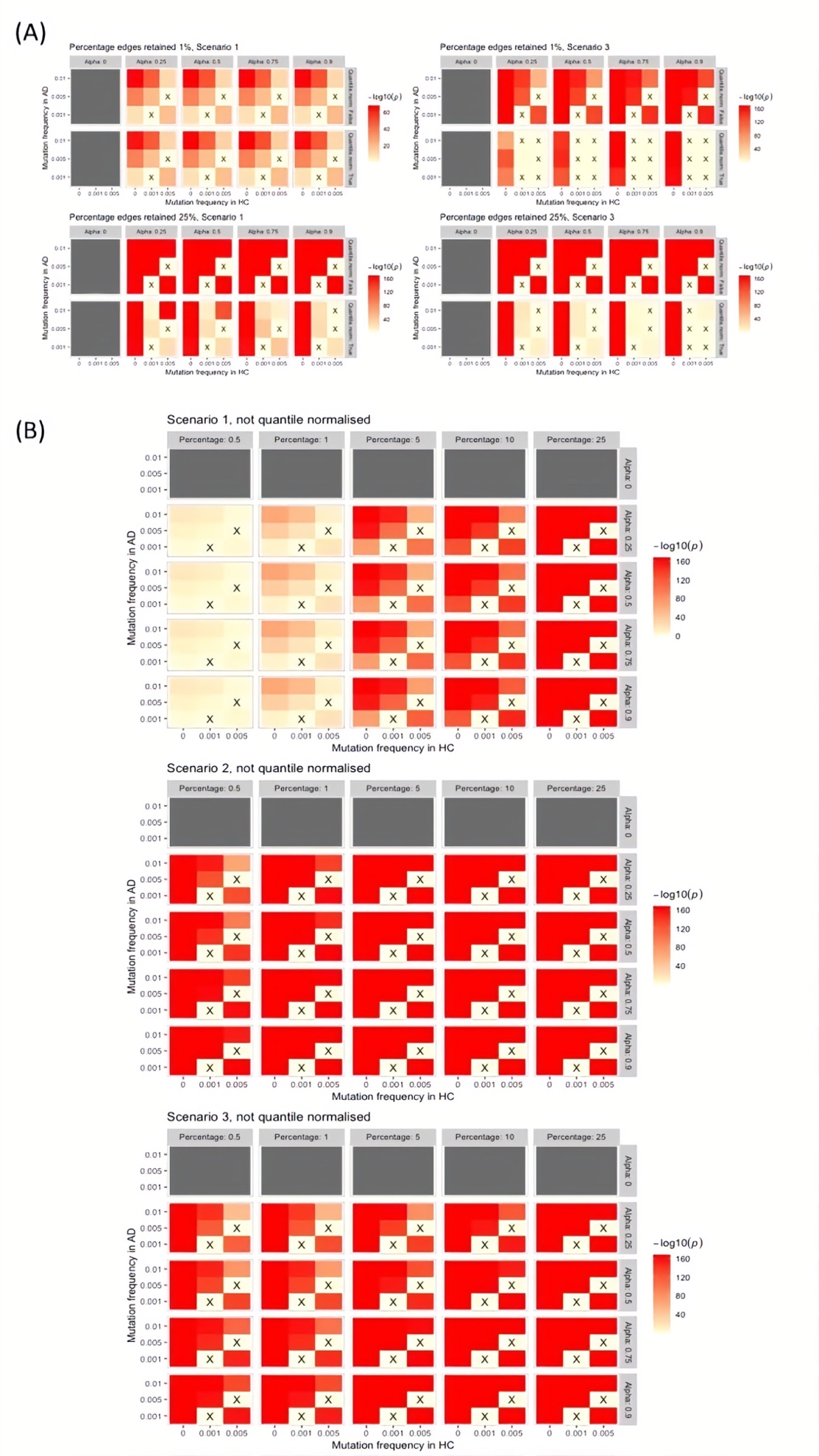

Supplement: S1 Fig — The x and y axis represent the mutation frequencies in controls and cases respectively. The faceting allows to visualise the effect of other parameters (diffusion length, percentage of edges retained, quantile normalisation). The colour-coding indicates the statistical significance of the difference in the hub gene smoothed score between controls and cases, in units of -log10 p-value. We investigated three mutation scenarios: scenario 1, only first neighbours mutated; scenario 2, only second neighbours mutated; scenario 3, both first and second neighbours mutated; the target gene is always unmutated. Cells marked with a black cross indicate parameter combinations where the smooth score of the hub gene was not significantly different between cases and controls. (A) Effect of quantile normalisation after network propagation with top 1% edges retained (top row, scenario 1 on the left, scenario 3 on the right), and with top 25% edges retained (bottom row, mutation scenarios as in top row). (B) Joint effect of percentage of edges retained and diffusion length for the three mutation scenarios considered. (DOCX) [file pcbi.1008517.s008.docx]
