# Supplementary material for: Network propagation of rare variants in Alzheimer’s disease reveals tissue-specific hub genes and communities
Source: PLoS Comput Biol. 2021 Jan 7;17(1):e1008517. doi: 10.1371/journal.pcbi.1008517 (PMC7817020; doi:10.1371/journal.pcbi.1008517)
Supplement: S2 Fig — In these stability paths plots, the x axis represents the steps taken along the lambda sequence to choose the optimal amount of regularisation required by the LASSO; the y axis represents the selection probability of a gene. Selection probability paths (trajectories) for different genes are represented by different colours. Genes whose trajectories crossed the threshold of 0.80 selection probability were considered as robust predictors of case-control status and followed up in subsequent analyses. The selection of PFAS is here replicated. (DOCX) [file pcbi.1008517.s009.docx]

**Supporting Information**

**Figure S2 -** Stability selection results in ADNI for a selection probability cutoff of 0.80, after propagating the burden of rare variants through the STRING and HuRI PPI networks. In these stability paths plots, the x axis represents the steps taken along the lambda sequence to choose the optimal amount of regularisation required by the LASSO; the y axis represents the selection probability of a gene. Selection probability paths (trajectories) for different genes are represented by different colours. Genes whose trajectories crossed the threshold of 0.80 selection probability were considered as robust predictors of case-control status and followed up in subsequent analyses. The selection of *PFAS* is replicated with STRING; *PFAS* is not present in HuRI, therefore it is not possible to replicate the finding.

**
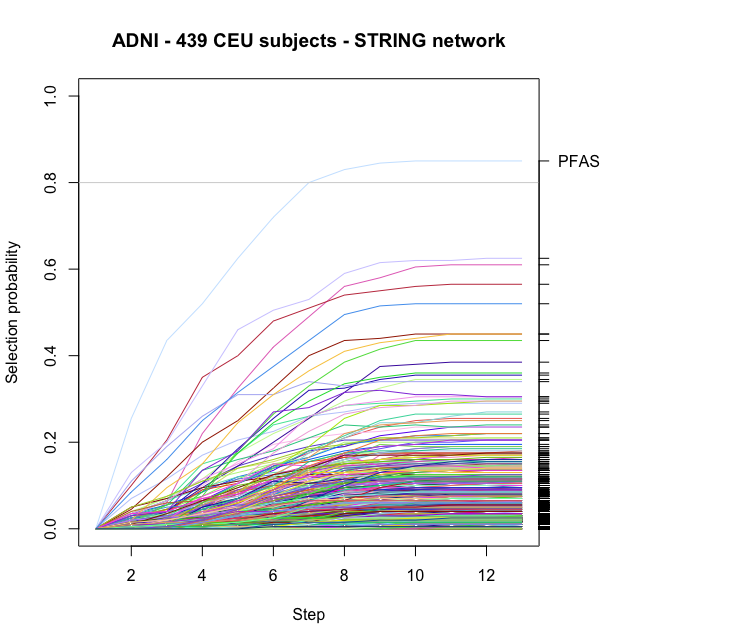
**

**
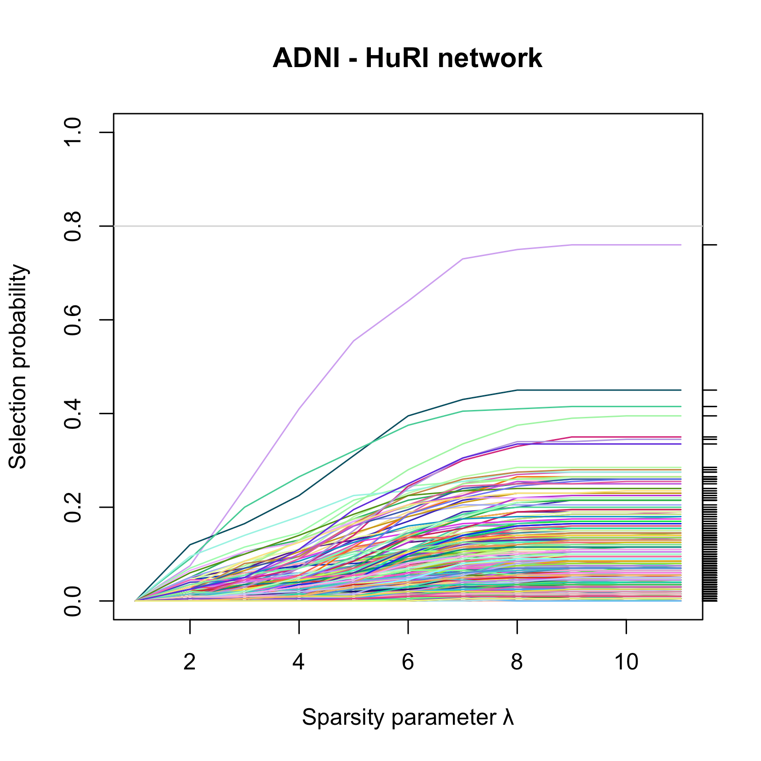
**
