# Supplementary material for: Network propagation of rare variants in Alzheimer’s disease reveals tissue-specific hub genes and communities
Source: PLoS Comput Biol. 2021 Jan 7;17(1):e1008517. doi: 10.1371/journal.pcbi.1008517 (PMC7817020; doi:10.1371/journal.pcbi.1008517)
Supplement: S3 Fig — Stability selection on (A) the raw (“unsmoothed”, α = 0) mutation profile in ADNI; (B) the mutation profile in ADNI smoothed through a randomised version of the hippocampus network; (C) the mutation profile in ADNI smoothed through a non-brain- related network (umbilical cord). No genes were selected in any of these negative controls with probability higher than 80%. (DOCX) [file pcbi.1008517.s010.docx]

**Supporting Information**


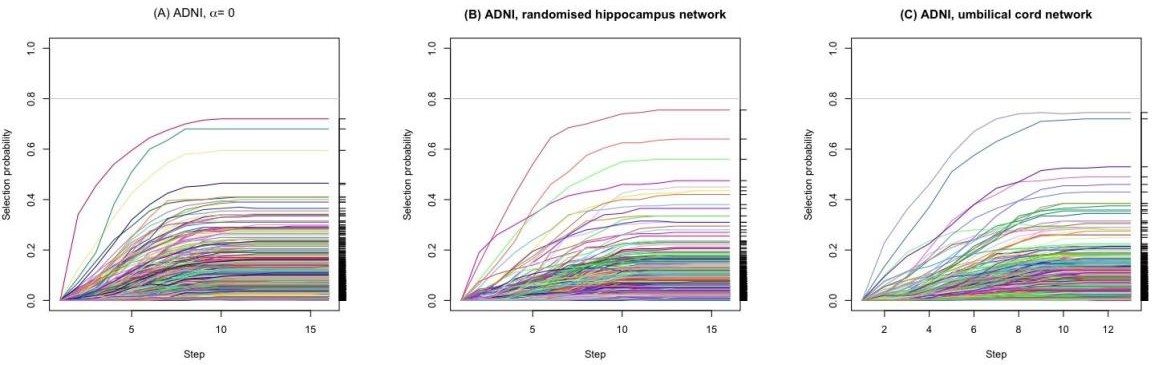
**Figure S3** - Stability selection on (A) the raw (“unsmoothed”, α = 0) mutation profile in ADNI; (B) the mutation profile in ADNI smoothed through one of 30 degree-preserving randomised versions of the hippocampus network; (C) the mutation profile in ADNI smoothed through a non-brain- related network (umbilical cord). No genes were selected in any of these negative controls with probability higher than 80%.
