# Supplementary material for: Network propagation of rare variants in Alzheimer’s disease reveals tissue-specific hub genes and communities
Source: PLoS Comput Biol. 2021 Jan 7;17(1):e1008517. doi: 10.1371/journal.pcbi.1008517 (PMC7817020; doi:10.1371/journal.pcbi.1008517)

**Supporting Information**

**Figure S4 –** Stability selection on brain structures and components (other than the hippocampus) from Greene et al.: top, ADNI WGS data smoothed through the neuron network; middle, ADNI WGS data smoothed through the medulla oblongata network; bottom, ADNI WGS data smoothed through the diencephalon network.


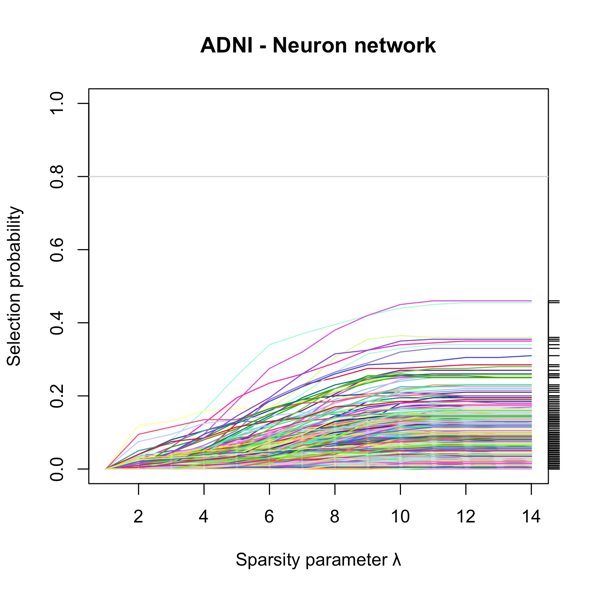


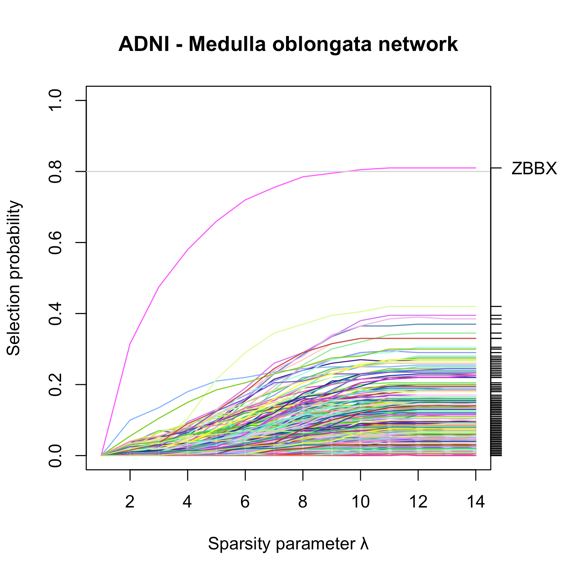


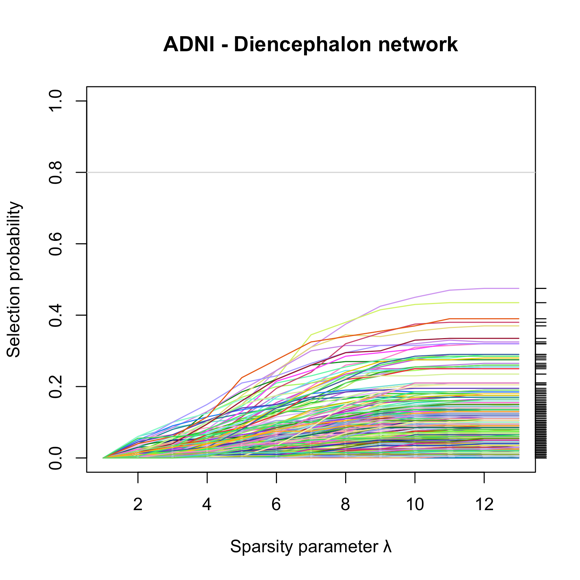

Supplement: S4 Fig — Stability selection on brain structures and components (other than the hippocampus) from Greene et al.: top, ADNI WGS data smoothed through the neuron network; middle, ADNI WGS data smoothed through the medulla oblongata network; bottom, ADNI WGS data smoothed through the diencephalon network. (DOCX) [file pcbi.1008517.s011.docx]
