# Supplementary material for: Network propagation of rare variants in Alzheimer’s disease reveals tissue-specific hub genes and communities
Source: PLoS Comput Biol. 2021 Jan 7;17(1):e1008517. doi: 10.1371/journal.pcbi.1008517 (PMC7817020; doi:10.1371/journal.pcbi.1008517)
Supplement: S5 Fig — Stability selection on a gene burden including only rare deleterious stop-gain, stop-loss and frameshift mutations, smoothed through (A) the hippocampus network from Greene et al (2015) (13,616 genes tested); (B) the STRING PPI network (13,385 genes tested). No genes were selected with probability higher than 80% in either of these alternative scenarios. (DOCX) [file pcbi.1008517.s012.docx]

**Supporting Information**

**Figure S5** - Stability selection on a gene burden including only rare deleterious stop-gain, stop-loss and frameshift mutations, smoothed through (A) the hippocampus network from Greene et al (2015) (13,616 genes tested); (B) the STRING PPI network (13,385 genes tested). No genes were selected with probability higher than 80% in either of these alternative scenarios.

**
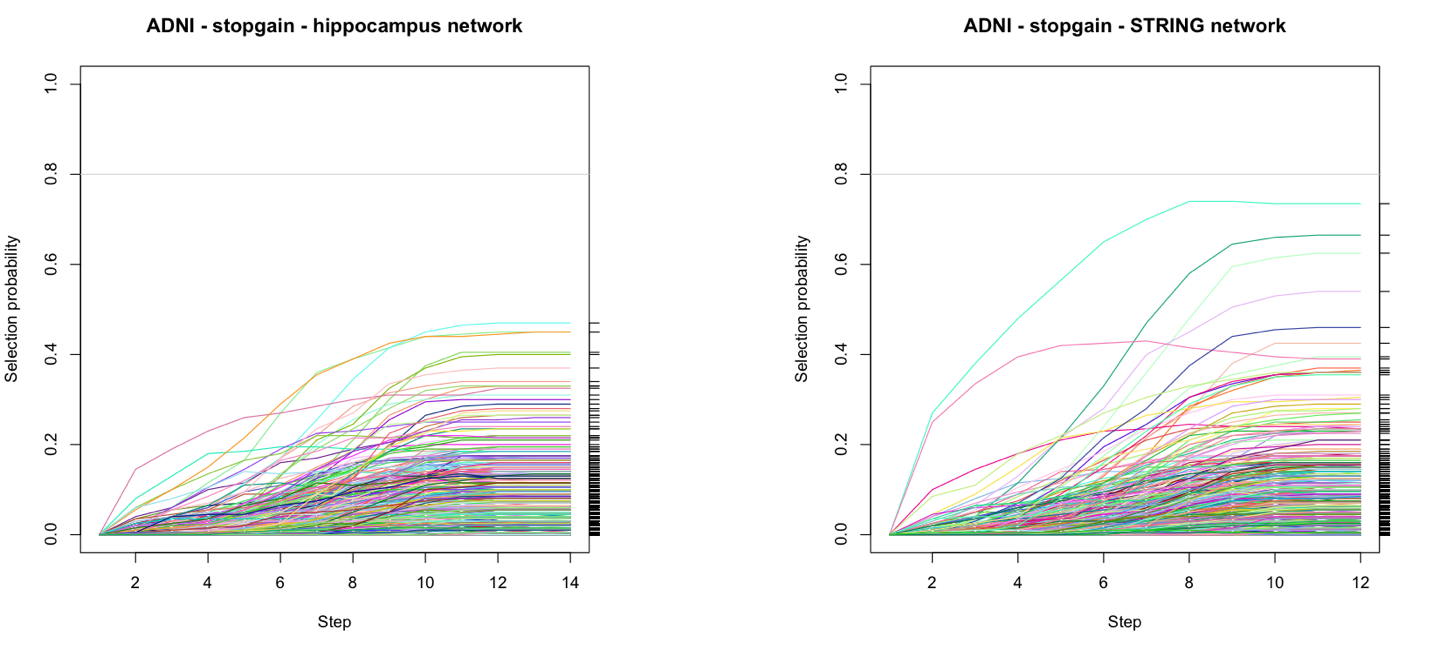
**
