# Supplementary material for: Network propagation of rare variants in Alzheimer’s disease reveals tissue-specific hub genes and communities
Source: PLoS Comput Biol. 2021 Jan 7;17(1):e1008517. doi: 10.1371/journal.pcbi.1008517 (PMC7817020; doi:10.1371/journal.pcbi.1008517)

**Supporting Information**

**Figure S6 -** Randomisation control over the amount of overlap between NETPAGE- selected genes in ADSP and the neighbourhood of PFAS. Eighteen out of 29 genes selected in ADSP were observed in the neighbourhood of PFAS (red vertical line). We formed 10,000 replicates of 29 genes selected randomly from the 16,298 genes tested, and counted how many genes in these replicates were also present in the neighbourhood of PFAS. The amount of overlap observed with the genes resulting from the ADSP experiment could not have been achieved by chance, but suggests the presence of functional connections linking NETPAGE’s results in the two independent datasets.


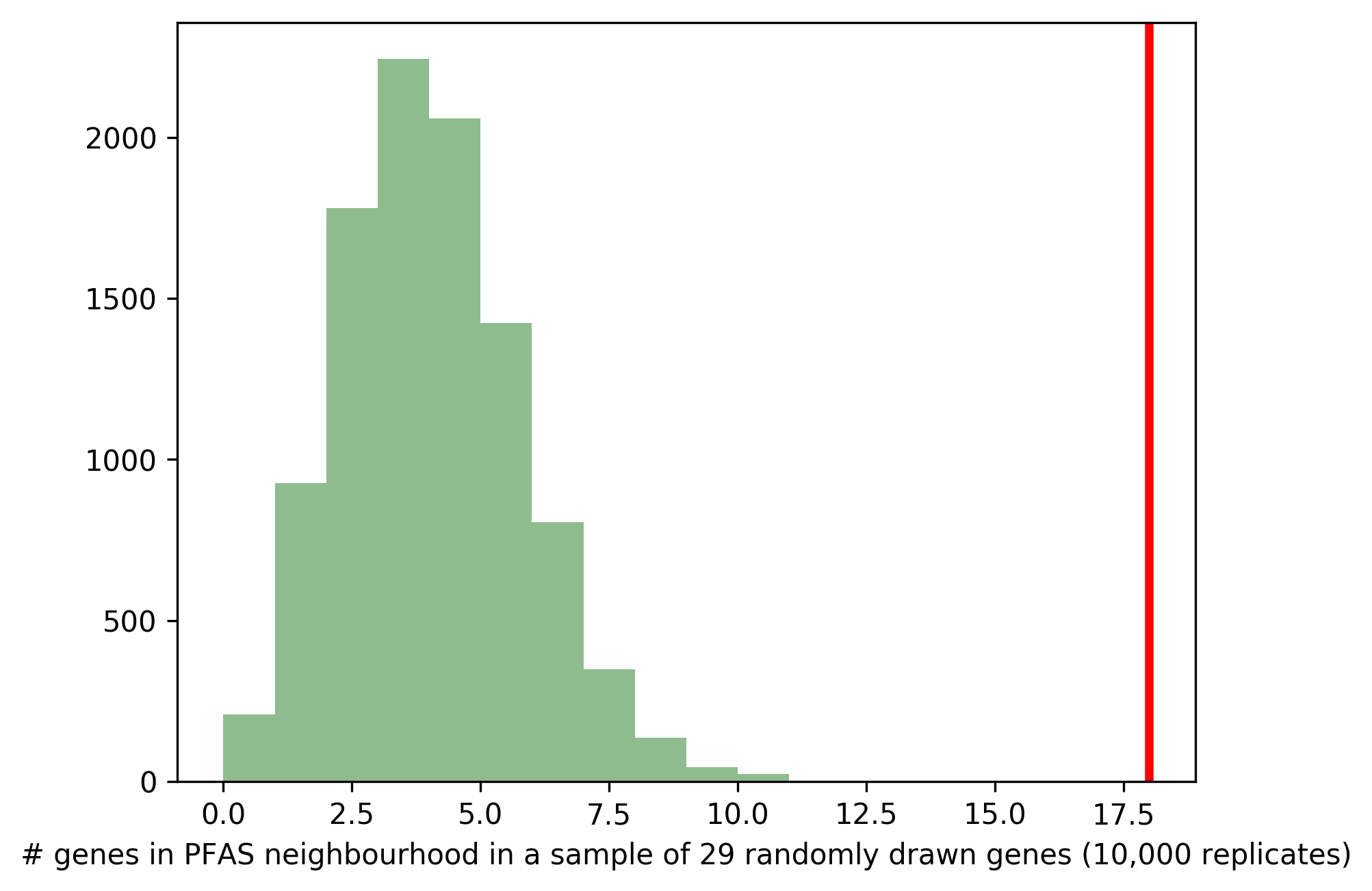

Supplement: S6 Fig — Eighteen out of 29 genes selected in ADSP were observed in the interactome of PFAS (red vertical line). We formed 10,000 replicates of 29 genes selected randomly from the 16,298 genes tested, and counted how many genes in these replicates were also present in the interactome of PFAS. The amount of overlap observed with the genes resulting from the ADSP experiment could not have been achieved by chance, but suggests the presence of functional connections linking NETPAGE’s results in the two independent datasets. (DOCX) [file pcbi.1008517.s013.docx]
