# Supplementary material for: Network propagation of rare variants in Alzheimer’s disease reveals tissue-specific hub genes and communities
Source: PLoS Comput Biol. 2021 Jan 7;17(1):e1008517. doi: 10.1371/journal.pcbi.1008517 (PMC7817020; doi:10.1371/journal.pcbi.1008517)

**Supporting Information**

**Figure S7** - Results of gene-based rare variant association testing in ADNI: (A) SKAT-O and (B) mass-univariate testing of smoothed scores against case-control status, in 439 Caucasian participants. Both tests were performed correcting for sex, age, years of education, number of *APOE* 4 alleles, and first two principal components population substructure.


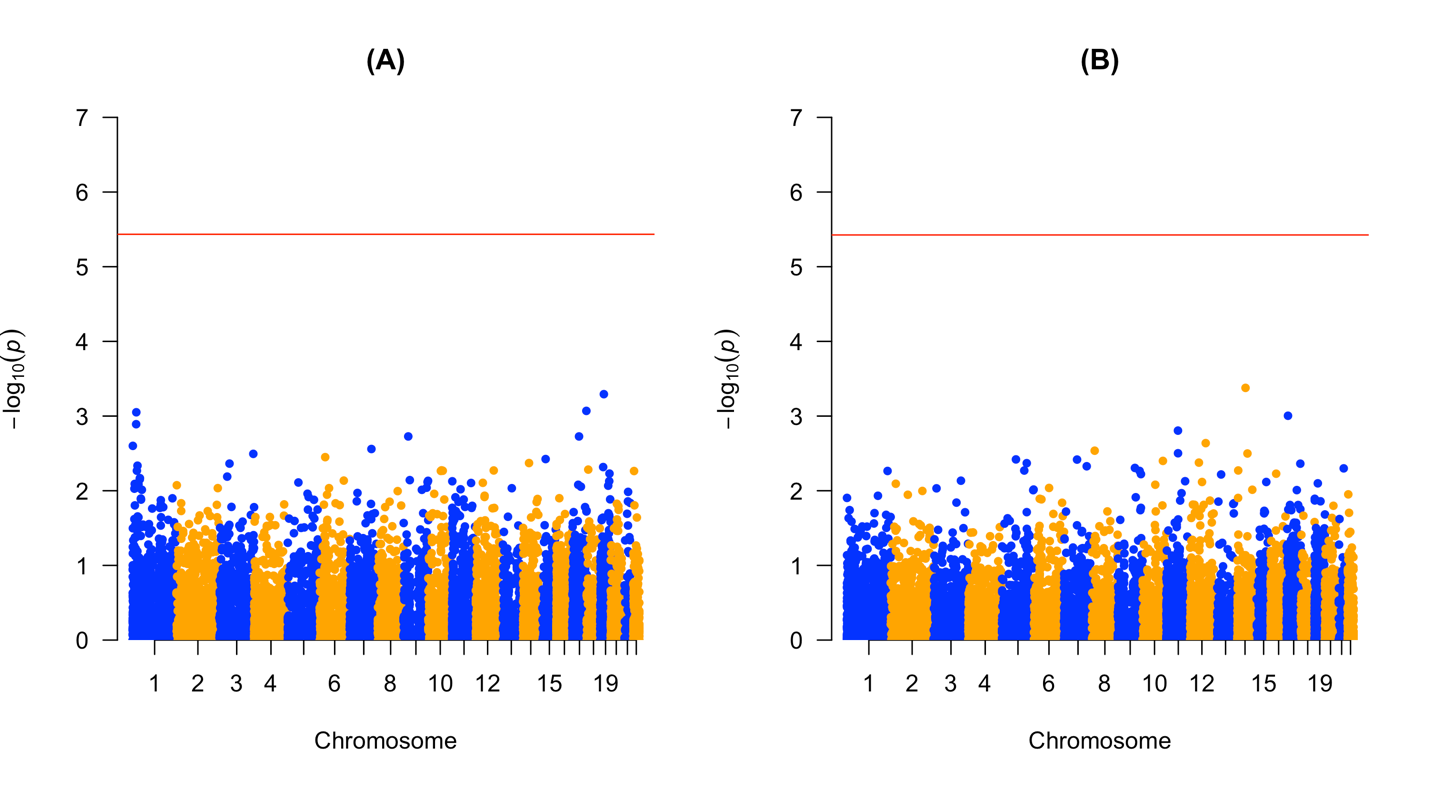

Supplement: S7 Fig — Results of gene-based rare variant association testing in ADNI: (A) SKAT-O and (B) mass-univariate testing of smoothed scores against case-control status, in 439 Caucasian participants. Both tests were performed correcting for sex, age, years of education, number of APOE 4 alleles, and first two principal components population substructure. (DOCX) [file pcbi.1008517.s014.docx]
