# Supplementary material for: Network propagation of rare variants in Alzheimer’s disease reveals tissue-specific hub genes and communities
Source: PLoS Comput Biol. 2021 Jan 7;17(1):e1008517. doi: 10.1371/journal.pcbi.1008517 (PMC7817020; doi:10.1371/journal.pcbi.1008517)

**Supporting Information**

**Figure S8** - Results of gene-based rare variant association testing (SKAT-O) in 10,186 unrelated individuals of Caucasian ancestry from ADSP (16,630 genes tested). The gene-wide significance threshold (red line) was set at 0.05/16,630 = 3x10^-6^. Gene-based models were corrected for sex, age, number of APOE ε4 alleles, and sequencing centre.


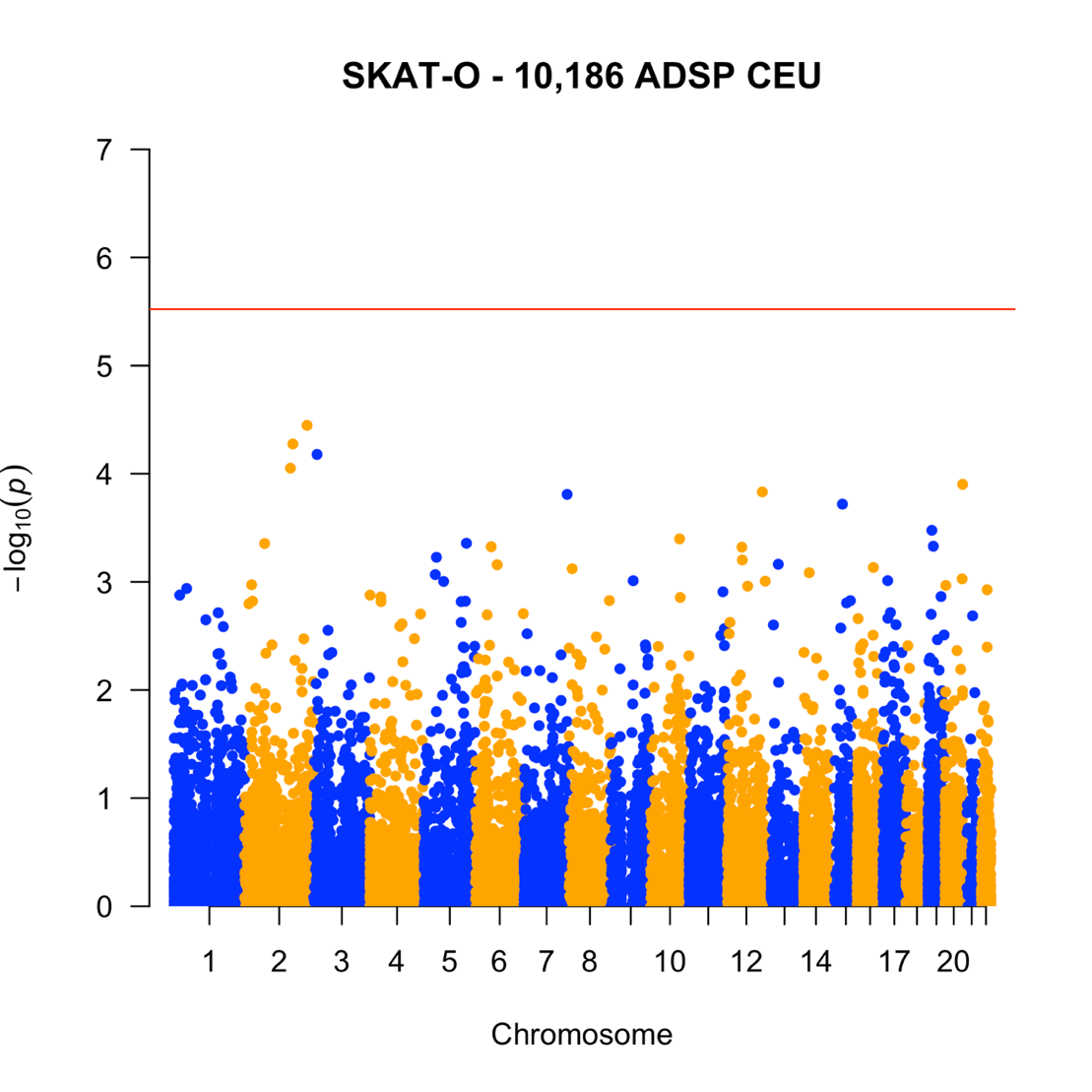

Supplement: S8 Fig — The gene-wide significance threshold (red line) was set at 0.05/16,630 = 3x10^-6^. Gene-based models were corrected for sex, age, number of APOE ε4 alleles, and sequencing centre. (DOCX) [file pcbi.1008517.s015.docx]
