# Supplementary material for: Network propagation of rare variants in Alzheimer’s disease reveals tissue-specific hub genes and communities
Source: PLoS Comput Biol. 2021 Jan 7;17(1):e1008517. doi: 10.1371/journal.pcbi.1008517 (PMC7817020; doi:10.1371/journal.pcbi.1008517)

**Supporting Information**

**Figure S9** - Survival analysis for the gene resulting from stability selection in ADNI. (A) Kaplan-Meier survival probability curves stratified by mutation status for *PFAS*. (B) The forest plot depicts hazard ratios from Cox proportional hazards model with confidence intervals and statistical significance. There was still no association between mutation status for PFAS and risk of conversion after covariate correction. (C) Results of the same Cox model fitting as in Figure 4, conducted after removing ADNI subjects diagnosed as cognitively normal at the latest time point available. Therefore the only samples where the conversion event did not occur used in this model were individuals with a stable diagnosis of MCI at the latest time point available. This aimed at partially avoiding circular analysis issues, as cognitively normal individuals were used as control samples in the discovery phase involving stability selection (Figure 3). The score resulting from network propagation for *PFAS* was still seen to be significantly associated with lower risk of conversion to AD after restricting to stable MCI the samples where the conversion event did not occur.


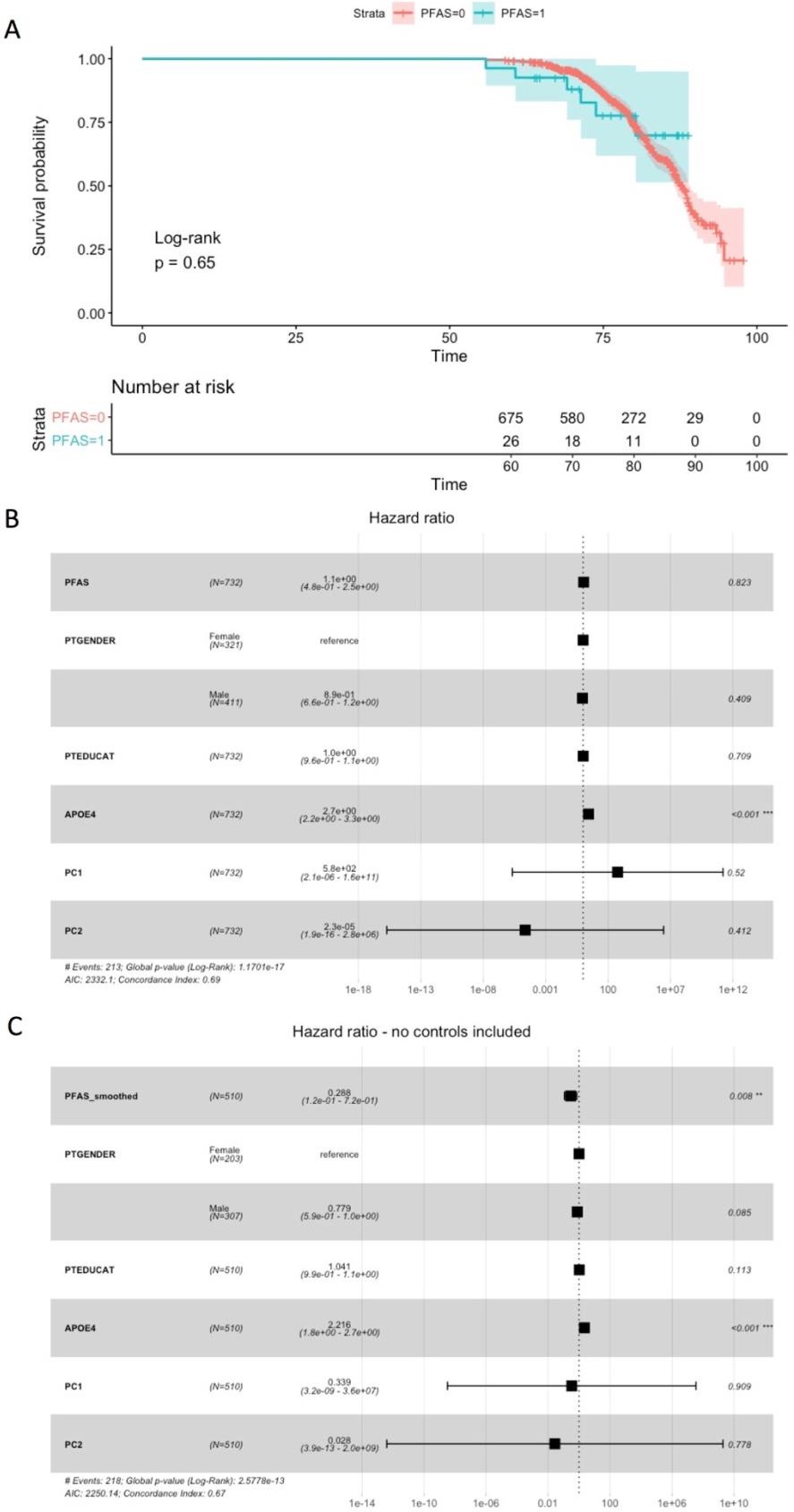

Supplement: S9 Fig — (A) Kaplan-Meier survival probability curves stratified by mutation status for PFAS. (B) The forest plot depicts hazard ratios from Cox proportional hazards model with confidence intervals and statistical significance. There was still no association between mutation status for PFAS and risk of conversion after covariate correction. (C) Results of the same Cox model fitting as in Fig 4, conducted after removing ADNI subjects diagnosed as cognitively normal at the latest time point available. Therefore the only samples where the conversion event did not occur used in this model were individuals with a stable diagnosis of MCI at the latest time point available. This aimed at partially avoiding circular analysis issues, as cognitively normal individuals were used as control samples in the discovery phase involving stability selection (Fig 3). The score resulting from network propagation for PFAS was still seen to be significantly associated with lower risk of conversion to AD after restricting to stable MCI the samples where the conversion event did not occur. (DOCX) [file pcbi.1008517.s016.docx]
