# Supplementary material for: Network propagation of rare variants in Alzheimer’s disease reveals tissue-specific hub genes and communities
Source: PLoS Comput Biol. 2021 Jan 7;17(1):e1008517. doi: 10.1371/journal.pcbi.1008517 (PMC7817020; doi:10.1371/journal.pcbi.1008517)

**Supporting Information**

**Figure S10 -** Reduction and visualisation in semantic space, through ReViGO, of the 928 GO terms significantly enriched (at p_FDR_ < 0.05) among the 1,449 first and second neighbours of PFAS in the hippocampus network. Top, ontology terms from GO Biological Process; middle, terms from GO Cellular Component; bottom, terms from GO Molecular Function. Bubble color indicates the term p-value (colour bar in lower right-hand corner); size indicates the frequency of the GO term in the underlying database (bubbles of more general terms are larger).


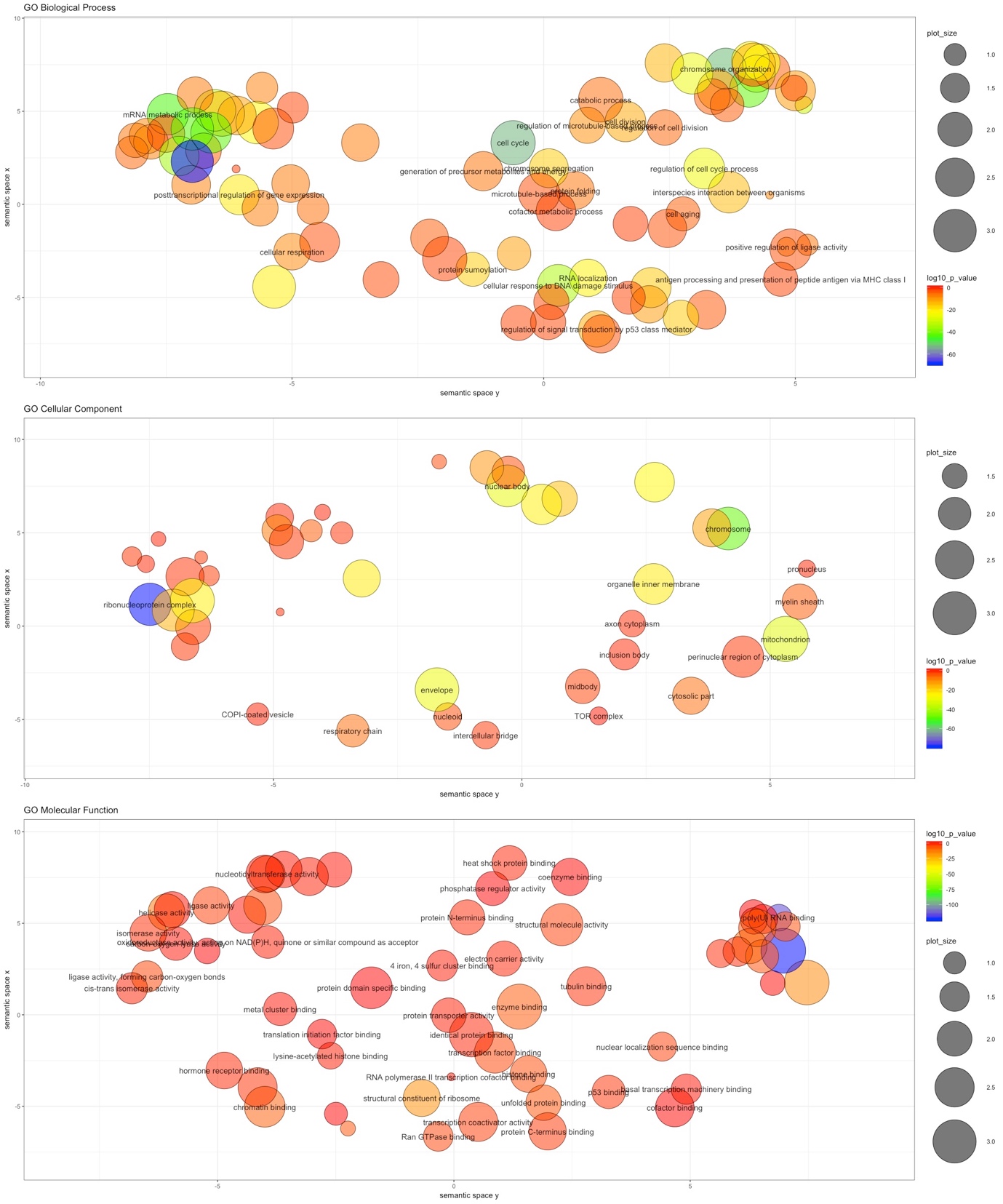

Supplement: S10 Fig — Top, ontology terms from GO Biological Process; middle, terms from GO Cellular Component; bottom, terms from GO Molecular Function. Bubble color indicates the term p-value (colour bar in lower right-hand corner); size indicates the frequency of the GO term in the underlying database (bubbles of more general terms are larger). (DOCX) [file pcbi.1008517.s017.docx]
