# Supplementary material for: Network propagation of rare variants in Alzheimer’s disease reveals tissue-specific hub genes and communities
Source: PLoS Comput Biol. 2021 Jan 7;17(1):e1008517. doi: 10.1371/journal.pcbi.1008517 (PMC7817020; doi:10.1371/journal.pcbi.1008517)

**Supporting Information**

**Figure S11** - Distributions of enrichment p-values for the seven AD-related CGP gene sets, and 1000 gene sets randomly sampled from the background; the red line indicates the location of the non-randomised, uncorrected p-values (P column in Table S2). We show that the significant overlap seen between our genes of interest and the AD-related gene sets curated by Blalock (Table S2) could not be achieved by chance, as none of the 1000 randomly drawn gene sets achieved smaller p-values.


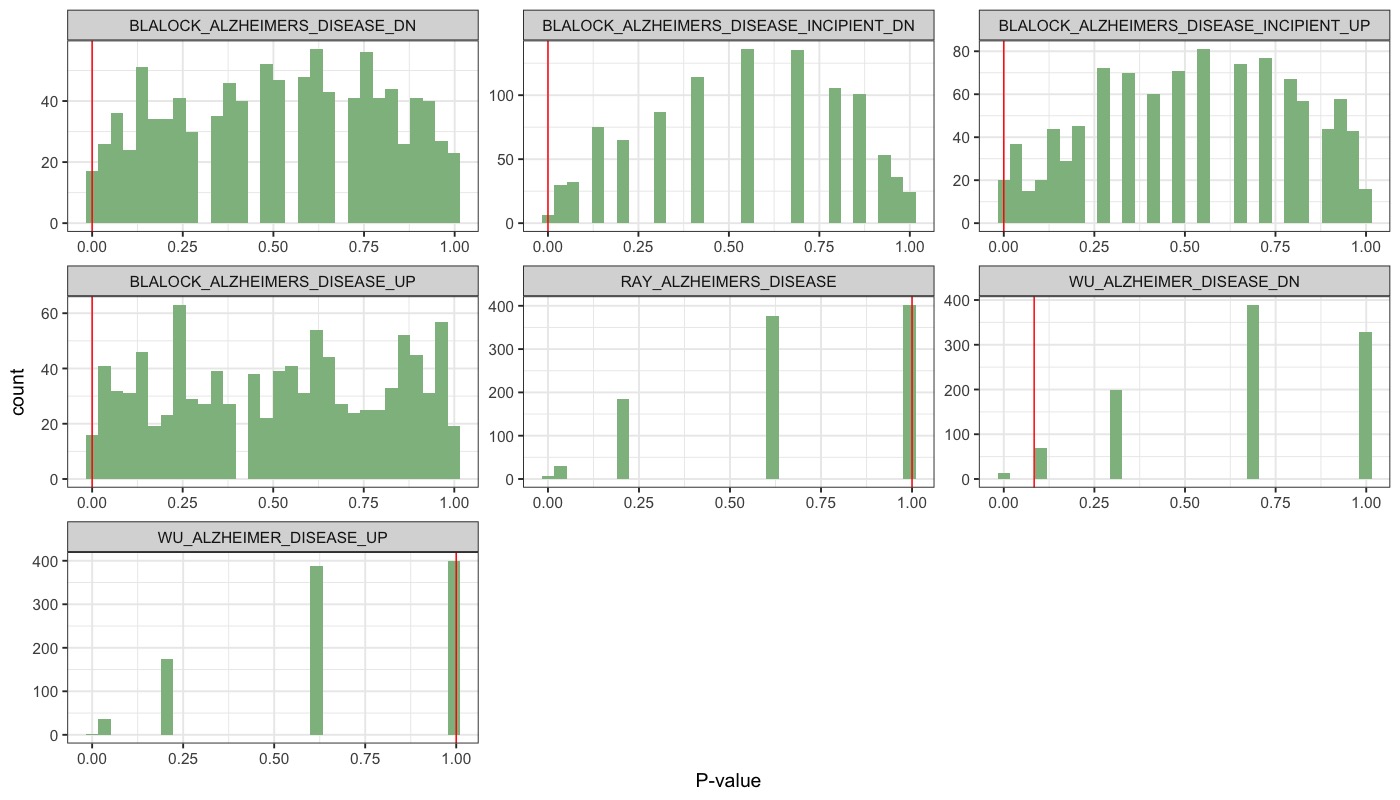

Supplement: S11 Fig — We show that the significant overlap seen between our genes of interest and the AD-related gene sets curated by Blalock (S3 Table) could not be achieved by chance, as none of the 1000 randomly drawn gene sets achieved smaller p-values. (DOCX) [file pcbi.1008517.s018.docx]
