# Supplementary material for: Network propagation of rare variants in Alzheimer’s disease reveals tissue-specific hub genes and communities
Source: PLoS Comput Biol. 2021 Jan 7;17(1):e1008517. doi: 10.1371/journal.pcbi.1008517 (PMC7817020; doi:10.1371/journal.pcbi.1008517)

**Supporting Information**

**Figure S12 -** Differential expression analysis for 30 selected genes in the Mount Sinai Brain Bank parahippocampal gyrus expression dataset. Full numerical results for all pairwise comparisons and their significance are provided in S5 Table.


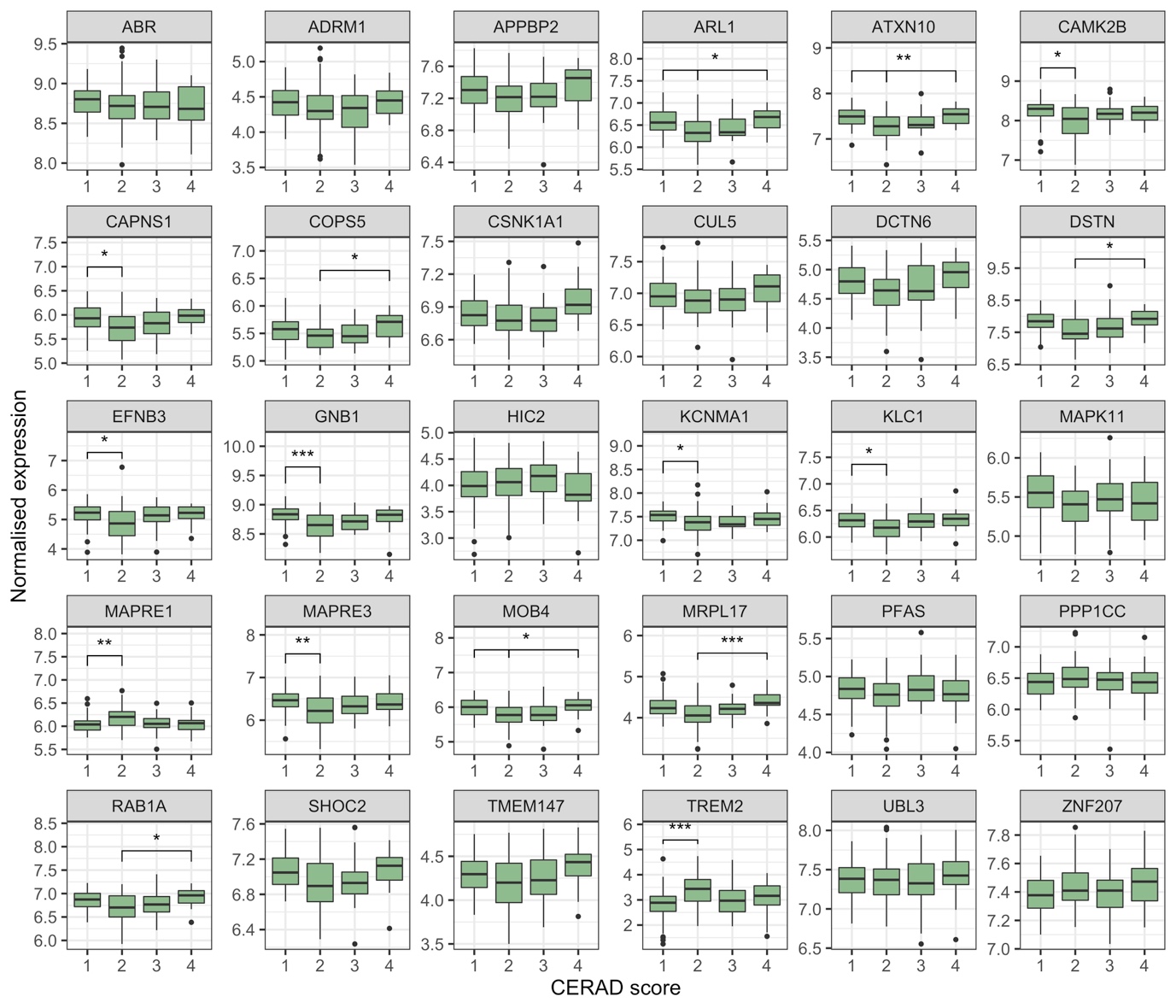

Supplement: S12 Fig — Full numerical results for all pairwise comparisons and their significance are provided in S4 Table. (DOCX) [file pcbi.1008517.s019.docx]
