# Supplementary material for: Network propagation of rare variants in Alzheimer’s disease reveals tissue-specific hub genes and communities
Source: PLoS Comput Biol. 2021 Jan 7;17(1):e1008517. doi: 10.1371/journal.pcbi.1008517 (PMC7817020; doi:10.1371/journal.pcbi.1008517)

**Supporting Information**

**Figure S13 -** Randomisation control over the differential expression analysis in the Mount Sinai Brain Bank dataset. We formed 1,000 sets of 30 randomly sampled genes and tested each of them for pairwise differences in expression levels among CERAD categories. We then counted how many genes in each set showed significantly different expression in at least one comparison and plotted their distribution. The red line indicates the 16 genes that showed significantly different expression in the original gene set of interest (i.e., the 30 genes selected in ADNI and ADSP). The data clearly shows that this amount of dysregulation could not be observed by chance, but is indeed linked to the overrepresentation of disease-related genes in the set of interest.


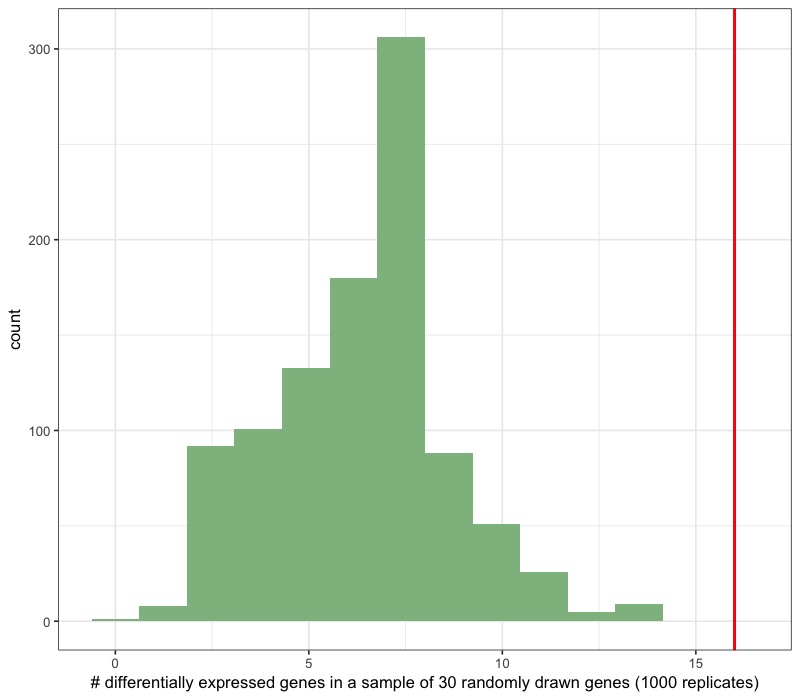

Supplement: S13 Fig — We formed 1,000 sets of 30 randomly sampled genes and tested each of them for pairwise differences in expression levels among CERAD categories. We then counted how many genes in each set showed significantly different expression in at least one comparison and plotted their distribution. The red line indicates the 16 genes that showed significantly different expression in the original gene set of interest (i.e., the 30 genes selected in ADNI and ADSP). The data clearly shows that this amount of dysregulation could not be observed by chance, but is indeed linked to the overrepresentation of disease-related genes in the set of interest. (DOCX) [file pcbi.1008517.s020.docx]
