# Supplementary material for: Network propagation of rare variants in Alzheimer’s disease reveals tissue-specific hub genes and communities
Source: PLoS Comput Biol. 2021 Jan 7;17(1):e1008517. doi: 10.1371/journal.pcbi.1008517 (PMC7817020; doi:10.1371/journal.pcbi.1008517)
Supplement: Attachment — Submitted filename: Rebuttal.docx [file pcbi.1008517.s021.docx]

Dear Professors Stelling and Elofsson,

On behalf of our colleagues we are pleased to submit this revision of our manuscript *“Network propagation of rare variants in Alzheimer’s disease reveals tissue-specific hub genes and communities”* for consideration as an original article at *PLOS Computational Biology*. We would like to thank you for the opportunity to revise our manuscript, and the reviewers for their expert feedback and suggestions.

As requested by the reviewers, we clarified the motivations that led us to not use the maximisation of prediction performance as a parameter optimisation strategy and why we resorted instead to simulation studies well calibrated on the current knowledge of loss-of-function genetic variation in the human genome. We also addressed the problem of degree-preserving randomisation of the network, and we studied in more depth the robustness of NETPAGE to different network sources and perturbations, as well as the connectivity properties of the novel disease genes identified. We find that the novel disease genes identified are network hubs and reside in dense communities connected by many back-up paths, in agreement with the disease module hypothesis; we also find that our method is robust to repeated instances of network randomisation and that, in agreement with previous work, the network architecture of Alzheimer’s disease is sensitive to perturbations and number of seed genes. Furthermore, we better placed our work in the context of existing literature on network propagation, highlighting similarities and differences with previous works, and deepened the discussion of the significance of our results.

Please find the point to point replies to the reviewers’ comments below. With these adjustments, we believe that our paper is decidedly stronger now. We hope you agree with all three reviewers that “*the statistical approaches used in this work are really interesting and the results are coherent and important*”, and that their presentation in the manuscript is “*well written and clear*”.

Sincerely yours,

Marzia Antonella Scelsi and Andre Altmann

========================================================================

**Reviewer #1**:

1. *Scelsi et al. have presented a new program, NETPAGE, of gene prioritization and have applied it to detect relevant genes associated with Alzheimer disease (AD). The program uses network propagation, and in this sense, it is not novel. However, they have used a very accurate protocol to infer the likelihood of their predictions, using bootstrap resampling and sparse regression selection; also, they have used a novel tissue-specific network to study the effect of rare deleterious genes associated with AD. The approach is very interesting and produces remarkable results. Nevertheless, I would need some extra explanations to clarify some details of their approach. [...] Overall, I think the statistical approaches used in this work are really interesting and the results are coherent and important, even if the method itself (or its basic principle) is not new.*

We are grateful to the reviewer for their thorough comprehension and very positive feedback on our work.

2. *There are some parameters, such as the mid-range α value of 0.5 and a top edge percentage of 1%, that need to be better explained, proving that they are optimal. For example, I would have expected the use of a 10 or 5 fold approach with known associated genes to optimise the prediction, taken the appropriate care on the use of a negative set (for example, I agree with some of the definitions used by the authors, randomising the network, although other approaches may also be valid).*

We thank the reviewer for raising a very good point. Indeed in the initial phases of method development, the optimisation of diffusion length and top edge percentage was based on a classic cross-validation framework to maximise AUC. However we faced two challenges with such an approach:

1. the absence of a gold standard, i.e., known associated genes. An appropriate gold standard in this situation would be a gene that, taken on its own, is NOT associated with Alzheimer’s disease (AD) through its burden of rare variants; but that becomes associated with AD when the burden of rare variants in its neighbouring genes is considered (where for neighbourhood we may go as far as the complete connected component). We are not aware of any results of the sort in the literature for AD genetics that could be used as gold standard - and that is actually why we undertook this study in the first place, to try and close this knowledge gap.
2. Complex trait prediction based solely on genetic markers is notably a difficult task that yields low accuracies [1]. This is why the prediction performance of highly-significant GWAS associations is never evaluated (for human traits). In this case, when we tried to apply cross-validation to maximise AUC, we saw discrimination performance stay relatively constant and no combination of parameters achieved significant improvements over the other combinations. This approach was therefore not informative for parameter optimisation.

Instead, to select the optimal values for the diffusion length α and the top edge percentage, we implemented a simulation framework that tries to maximise the separation (effect size) between cases and controls in the smoothed score of the core gene, while retaining network connections with the highest confidence (edge weights); we also integrated previous knowledge about the behaviour of network propagation, particularly the work in [2] where it is shown that the specific value of α has a minor effect on the results of network propagation over a sizable range (0.5–0.8). The only parameter that is left to be tuned with the classic approach of optimising prediction (even though using stability selection rather than cross-validation) is the LASSO penalty λ. We added to the manuscript (page 8, lines 178-180) a sentence to summarise why we chose simulations over optimisation of cross-validated performance: “*We chose to implement a simulation framework for this task because we found the standard approach of optimisation of cross-validated prediction performance not informative in this setting.*”

[1] [A Foundation for Provitamin A Biofortification of Maize: Genome-Wide Association and Genomic Prediction Models of Carotenoid Levels](https://www.genetics.org/content/198/4/1699.short)

[2] [Network-based stratification of tumor mutations](https://www.nature.com/articles/nmeth.2651)

3. *Some relatively recent or well established works on networks have used other approaches, instead of selecting a fixed top percentage threshold. For example, the functional-based selection of top-ranking genes, which is a procedure to identify if the set of ranking genes is functionally similar to the set of initial seed genes. This procedure was implemented in Ghiassian et al. [1]in DIAMOnD. The authors should proof that the selection of 1% top-ranking genes produces acceptable results (only as an example, Sharma et al. showed that DIAMOnD outperformed existing algorithms in predicting asthma related genes[2], the authors may not need to proof their approach is better, but using a threshold of 1% should be at least compared with other values or criteria).*

We recognise that selecting a fixed top percentage threshold of edges is one of many possible ways to tackle network complexity.

Firstly, we would like to clarify that the ranking in our approach does not apply to network nodes (genes, as in the case of DIAMOnD), but to network edges. The networks used as substrate for network propagation (GIANT and STRINGdb) are weighted networks, therefore the ranking of edges in terms of these weights is an intrinsic property of the network and is intended to quantify the strength of evidence for a given connection between two genes. This is a feature of the dataset and not generated by NETPAGE, in contrast to what DIAMOnD does (i.e., ranking genes by their connectivity significance).

We would also like to expand on the rationale that led us to the use of fixed top percentage of edges: the available networks are weighted, whereas the mathematical formulation of network propagation requires an adjacency matrix of 0s and 1s (unweighted network). For this reason, our pipeline performs a step of network “binarization”, i.e., setting to 1 the top-weights edges and to 0 all the others, effectively removing low-weight edges from the interactome and generating incompleteness in the underlying graph. If unweighted networks are used (such as the interactome by Luck et al. suggested by the reviewer), there is no need to perform such binarization. However, in contrast to Ghiassian et al., this network binarization procedure does not affect the number of “seed” genes; in our case a gene is a seed for network propagation if and only if it contains rare deleterious mutations, independently from the number of edges incident on that seed gene.

Another difference with the DIAMOnD algorithm is that the initial seed genes for DIAMOnD are chosen based on known associations reported in OMIM and GWAS (hence on **common** variants association results); whereas here we label a seed gene if it contains any **rare deleterious** variant, irrespective of whether an association was previously reported, because the knowledge in this area of Alzheimer’s disease is scarce and we did not want to limit ourselves.

But we do agree with the reviewer that the use of a percentage of top edges is yet another degree of freedom in the method and can introduce variability in the results. For this reason we used simulations to understand how the percentage of top edges retained affects network propagation and to select a default value of 1% (Supplementary Figure 1A-B). Here we varied this percentage between 0.5% and 25% and noticed that in all cases the smooth score for the hub gene provides essentially the same separation between cases and controls. Therefore, increased incompleteness in the graph substrate has a negligible effect on the spread of mutation signals through the network, and we believe this is due to the underlying high density of connections and small-worldness of the biological networks utilised.

4. *Finally, the authors have tested the robustness of their approach. Specifically, the sentence is: “Lastly, we investigated the robustness of the method with respect to the network structure and derivation methods by using the human, non- tissue-specific protein-protein interaction network (PPI) available through the STRING database”. I think there are some important issues for this analysis:*

*1) using the STRING database was an excellent approach when they decided to analyse a functional network, but it is not the right choice to analyse the protein interaction network. I would suggest using the recently published network of the human interactome by Luck et al. [3].*

We adapted the code and ran NETPAGE using the human interactome by Luck et al. (HuRI, downloaded from <http://www.interactome-atlas.org/data/HuRI.tsv> ) as a substrate for network propagation. Here the main features of this graph:

This graph contains non-tissue-specific, high-quality, curated protein-protein interactions. Nodes are labelled with their Ensembl Gene identifier. As a result of the curation and the emphasis on high-quality interaction data, the number of network nodes is relatively low (8k genes) compared to STRING or to the tissue-specific networks compiled by Greene et al (16k genes). Of these 8k genes, only 5,351 are in common with the ADNI annotated whole genome sequencing data. Importantly, *PFAS* (ENSG00000178921) is not present in HuRI, therefore it is not possible to replicate the finding we originally presented. Nevertheless, we ran NETPAGE on ADNI using HuRI as the reviewer suggested, and no genes were selected as robustly associated with Alzheimer’s disease diagnosis:

The only gene approaching the 80% selection probability threshold is *AIFM2* (lilac trajectory in the plot above). This is a pro-apoptotic gene that is connected to *PFAS* in STRINGdb through *TP53*, but the connection has a low confidence score (< 0.4, see graph below where the line thickness indicates the strength of data support). The low confidence in this interaction may explain why there is no path linking *AIFM2* and *PFAS* neither in the hippocampus functional network nor in HuRI and why, when using the latter network, the mutation signal propagated through the network and stopped/converged on *AIFM2*. We conclude that, despite being influenced by the number of network nodes and connections, the presented method is robust to network structure because it allows mutation signals to follow different pathways but to converge always in the neighbourhood of the same genes.

We have included a reference to this additional test of robustness with HuRI and its results in the Methods section and in Supplementary Figure 2.

*2) Some other works have tested the robustness of network propagation approaches for gene-prioritization [4] and the relevance of the network completion (i.e. using other near or proximal neighbouring search approaches [5]). Interestingly, not all diseases have a network connectivity that allows for the use of network propagation approaches to predict gene-disease associations or infer relevant pathways. Therefore, the robustness and network-features for this study should be more deeply studied, above all for its dependency on the network and on the network completion (i.e. analyses on the connectivity of gene-disease associations depending on the network, testing artificially the effect of edge removal or the dependency on the number of seeds used for the propagation).*

The reviewer signals three aspects of our work that should be more deeply studied. We address each of them below.

a) “*analyses on the connectivity of gene-disease associations depending on the network”:* In response to a similar comment made by reviewer #2, we looked at the connectivity of disease-associated genes in terms of their degree in the hippocampus functional network. We would like to refer the reviewer to our response to comment #4 from reviewer 2, and the associated table that we also included in the manuscript as Supplementary Table 3. From this analysis we conclude that all the selected genes are hubs of the hippocampus functional network. The average degree of the network is 22, therefore all these genes are hubs in the sense that their degree greatly exceeds the average. When we look at the degree of the same disease-associated genes in other networks (see below), a diverse picture arises. Firstly, not all disease-associated genes are present in all networks: this is because STRING, HuRI, and the tissue networks from Greene et al. were derived with different curation pipelines and therefore in their original form (as we downloaded them from the respective databases) these networks comprise different sets of genes. Then, in comparison to the respective network average degree, some genes can be identified as hubs while most of them are not. This allows us to conclude - in alignment to our response to comment #3 from reviewer 2 - that gene-disease associations are not solely influenced by the degree of the gene, but that the underlying gene interaction network and tissue-specificity are indeed contributing to detecting disease associations.

In the following we list the degree of disease genes, as identified in the Greene hippocampus network, in other networks, to give an example of how the connectivity properties of these genes change according to the network/tissue used:

- **STRING**: average degree 4.17. Degree of disease-associated genes: CAPNS1: 6, HIC2: 0, ARL1: 7, KCNMA1: 2, CAMK2B: 9, TMEM147: 1, ABR: 0, TREM2: 3, KLC1: 12, ADRM1: 24, SHOC2: 3, PFAS: 5.
- **HuRI**: average degree 8.18. Degree of disease-associated genes: TMEM147: 30, KLC1: 4, HIC2: 3, CAMK2B: 16.
- **Greene et al., neuron**: average degree 33.75. Degree of disease-associated genes: CAPNS1: 99, HIC2: 34, ARL1: 82, KCNMA1: 213, CAMK2B: 77, TMEM147: 71, ABR: 30, TREM2: 12, KLC1: 114, ADRM1: 61, SHOC2: 24, PFAS: 31.

Another possible approach to characterise the connectivity of disease-associated genes is to look at their pairwise (or local) node connectivity. Pairwise or local node connectivity between two distinct and nonadjacent nodes is the minimum number of nodes that must be removed (minimum separating cutset) to disconnect them. The figure below shows the matrix of pairwise connectivities for the 30 selected genes in the hippocampus network in the form of a heatmap. Consistently with the disease module hypothesis (*), the disease genes identified by NETPAGE tend to reside in a tightly interconnected community, to the point that to disconnect some pairs of genes it is necessary to remove at least 400 other genes from the network. A special case in this network is TREM2, which in this binarised network appears to be an isolate. For an explanation of why this happens we refer the reviewer to our response to comment #4 from Reviewer 2.

(*) Barabási AL, Gulbahce N, Loscalzo J. Network medicine: a network-based approach to human disease. *Nat Rev Genet*. 2011;12(1):56-68. doi:10.1038/nrg2918

b) *“testing artificially the effect of edge removal”:*

To address this comment we implemented a new option whereby, after picking a top percentage of edges, the user can choose to perform **probabilistic edge removal**, i.e., for each gene removing with a given probability a random edge. For this option we adapted some publicly available python code (<https://stackoverflow.com/questions/42591549/add-and-delete-a-random-edge-in-networkx>). We tested this new option with a probability of 0.1 (--edgeremoval --probremoval 0.1).

As expected, edge removal is a type of perturbation that results in decreased connectivity of the network overall, with the number of isolated nodes increasing from 5761 to 5886 and the number of connected components also increasing from 5782 to 5904. When testing the gene scores obtained from propagation through this perturbed network for association with Alzheimer’s disease diagnosis, we did not replicate the selection of *PFAS* (nor identify any other gene). This result is in agreement with the findings in Guney and Oliva (2014; Table 1 in reference #4 provided by the reviewer): in this paper the authors demonstrate that the network structure of Alzheimer’s disease is not robust (“non-tolerant”) to perturbations like edge removal or edge swapping.

c) “*dependency on the number of seeds used for the propagation*”: The dependency on the number of seeds used for propagation was investigated when we looked at different rare SNV inclusion criteria to derive a gene mutational status. The reviewer will recall that we constructed a second set of SNVs, by retaining only rare variants predicted to have a stop-gain, stop-loss or frameshift consequence with a CADD score > 20 (please see the *Methods: Variant filtering and mapping* section). Because these SNVs have a higher functional impact on the resulting proteins, they are subject also to higher negative selection and therefore found in much fewer genes (in comparison to the generic non-synonymous variants that we selected initially). Indeed, this second set comprised 4,061 SNVs mapped to 2,910 genes (instead of the 48,834 SNVs and 13,591 genes mapped initially). Therefore, with this second set the number of seed genes (i.e., genes where such SNVs can be found) used for propagation is reduced almost five-fold. We showed that NETPAGE is sensitive to such a reduction in the number of propagation seeds, as no genes are selected in this second case study, irrespective of the network used (Supplementary Figure 5). We attributed this behaviour to the resulting much sparser SNV profiles, to the point that the signal amplification property of network propagation is no longer enough to reveal meaningful patterns. Finally, we comment in the Discussion section that “the inclusion criteria for SNVs – which are not to be regarded as a parameter of the algorithm but rather as a user choice – need to reflect careful consideration of the disease area under study as well as the experimental conditions”.

*References*

*1. Ghiassian SD, Menche J, Barabasi AL. A DIseAse MOdule Detection (DIAMOnD) algorithm derived from a systematic analysis of connectivity patterns of disease proteins in the human interactome. PLoS Comput Biol. 2015;11(4):e1004120. doi: 10.1371/journal.pcbi.1004120. PubMed PMID: 25853560; PubMed Central PMCID: PMCPMC4390154.*

*2. Sharma A, Menche J, Huang CC, Ort T, Zhou X, Kitsak M, et al. A disease module in the interactome explains disease heterogeneity, drug response and captures novel pathways and genes in asthma. Hum Mol Genet. 2015;24(11):3005-20. Epub 2015/01/15. doi: 10.1093/hmg/ddv001. PubMed PMID: 25586491; PubMed Central PMCID: PMCPMC4447811.*

*3. Luck K, Kim D-K, Lambourne L, Spirohn K, Begg BE, Bian W, et al. A reference map of the human binary protein interactome. Nature. 2020. doi: 10.1038/s41586-020-2188-x.*

*4. Guney E, Oliva B. Analysis of the robustness of network-based disease-gene prioritization methods reveals redundancy in the human interactome and functional diversity of disease-genes. PLoS One. 2014;9(4):e94686. Epub 2014/04/16. doi: 10.1371/journal.pone.0094686*

*PONE-D-14-06874 [pii]. PubMed PMID: 24733074.*

*5. Menche J, Sharma A, Kitsak M, Ghiassian SD, Vidal M, Loscalzo J, et al. Disease networks. Uncovering disease-disease relationships through the incomplete interactome. Science. 2015;347(6224):1257601. doi: 10.1126/science.1257601. PubMed PMID: 25700523; PubMed Central PMCID: PMCPMC4435741.*

========================================================================

**Reviewer #2**:

1. *The authors have provided a good approach for network propagation of rare variants, following a natural extension of the established literature.*

We thank the referee for their positive evaluation of our work.

2. *However, I have significant concerns, especially in the area of the strength of their network controls. Specifically, the authors have not convinced me that they are correctly controlling for the network topology; in particular, I would like to see their tests performed on null networks that approximately preserve the degree of all genes but scramble the identity of edges.*

This is indeed a very important point and we are grateful to the referee for flagging it. We have now updated the code for network propagation to perform degree-preserving randomisation when the --randomnet flag is present, adapting a publicly available python implementation by C. Lasher (<https://gist.github.com/gotgenes/2770023#file-edgeswap-py>). We also introduced a --nrand flag that allows the user to specify how many randomised replicates of the network should be generated and used for network propagation. This will be useful to address the following comment.

3. *Although you perform a single test against a randomized version (which should be degree-preserving, so that genes of high degree stay high degree, simple edge shuffling will destroy this relationship) of the network for Figure S3, there is not enough statistical power for me to be sure that this is well controlled. A test of 30 iterations or so (enough to get a variance estimate on the number of associated genes) should show that gene association is not solely influenced by the degree of the genes that are selected, and would convince me that the real underlying network is contributing to detecting disease association.*

As mentioned in the previous comment, we have modified the source code of NETPAGE to generate a user-defined number of randomised replicates of the gene interaction network and to run network propagation on all of them. When conducting sparse regression on the resulting 30 sets of smoothed gene scores, no gene was selected from any of the 30 sets. Please find below the stability selection path plots for these 30 test iterations. The two genes that came close to the threshold in randomisations 2 and 18 are *DIMT1* and *PSMA6*. We believe this result provides convincing evidence that gene associations are not solely influenced by the degree of the selected genes, but that the underlying gene interaction network is indeed contributing to detecting disease association. We have included an explicit reference to the fact that the randomised networks were degree-preserving, acknowledged the existing implementation leveraged in the source code, and reported the results in the Methods and Results (“*Small sample: ADNI”*) sections, as well as in Supplementary Figure 3.

4. *I would also like to see you address the underlying question of degree directly; is there a bias toward hubbier genes? Are hubby genes that have initial score more or less contributing to the final association than sparsely connected genes?*

Yes, as mentioned in the manuscript title, we find that all the selected genes are hubs of the hippocampus functional network (see table below for their degrees). The average degree of the network is 22, therefore all these genes are hubs in the sense that their degree greatly exceeds the average. We envisage that future method developments may include an alternative test, where the score from network propagation is compared to an expected score based on gene degree in the network (or a test that builds on this idea). This is a point that we will definitely investigate in future work, but outside the scope of this revision; therefore we spell it out more clearly in the discussion that this will be the object of future investigation. However, after analysing the behaviour of 30 degree-preserving randomised networks as previously suggested by the reviewer, we feel confident that gene associations are not solely influenced by the degree of the selected genes, but that the underlying gene interaction network is indeed contributing to detecting disease association.

The only exception to the hub property of selected genes is *TREM2*, which appears to be an isolated node. The apparent disconnection of *TREM2* from the rest of the network is due to the network binarisation procedure that removes edges outside a chosen top percentage of ranked weights. However, despite being disconnected from the network, and therefore not receiving any signal from neighbouring genes, *TREM2* was still identified by stability selection as a predictor of AD diagnosis. Essentially what happened in this case is that the mutation status (presence or absence of rare nonsynonymous mutations) of TREM2 was discriminative enough to be recognised by stability selection as a robust predictor *per se*, despite not benefitting from the enhancing properties of network propagation due to the lack of links to the rest of the network. This appears reasonable based on the extensive knowledge currently available about the impact of rare coding mutations in *TREM2* on AD risk [Guerreiro 2013, Jonsson 2013, Jin 2014, Song 2016, Ulrich 2017].

If it’s reasonable to expect an association signal for TREM2, the reviewer may be wondering why none was found when running the SKAT-O test on ADSP (Supplementary Figure 8). We take here the opportunity to clarify a difference that in our opinion explains the discordant result. SKAT-O looks at the cumulative burden of rare variants collapsed into a single gene score: for this reason, it is more sensitive to the criteria for rare variants inclusion than our approach, which defined a simple binary variable denoting presence or absence of rare variants. Further details are given in the section of Discussion relative to benchmarking, also with respect to existing literature.

| Gene | Degree | Gene | Degree |
| --- | --- | --- | --- |
| *ABR* | 285 | *KCNMA1* | 297 |
| *ADRM1* | 299 | *KLC1* | 403 |
| *APPBP2* | 182 | *MAPK11* | 76 |
| *ARL1* | 361 | *MAPRE1* | 227 |
| *ATXN10* | 105 | *MAPRE3* | 144 |
| *CAMK2B* | 219 | *PFAS* | 41 |
| *CAPNS1* | 207 | *MOB4* | 270 |
| *COPS5* | 389 | *MRPL17* | 80 |
| *CSNK1A1* | 278 | *PPP1CC* | 298 |
| *CUL5* | 159 | *RAB1A* | 382 |
| *DCTN6* | 135 | *SHOC2* | 365 |
| *DSTN* | 163 | *TMEM147* | 339 |
| *EFNB3* | 207 | *TREM2* | 0 |
| *GNB1* | 412 | *UBL3* | 154 |
| *HIC2* | 153 | *ZNF207* | 190 |

5. *Along these lines, the ADSP set should be run on the alternative networks derived from other tissues. I see no indication that tissue specificity of the networks is contributing to better results. In the case of ADNI, you find a single gene association, PFAS, whereas running on the medulla oblongata net finds one as well, ZBBX. Is there any reason to indicate that the tissue specificity helps? Again, a series of randomized controls can help here, since the correct tissue specific network should outperform its corresponding null networks better than the incorrect tissue, but it needs a statistical argument.*

We would like to clarify that, even though we stress the importance of tissue specificity, there is no “correct” or “incorrect” tissue network in an absolute sense, but the relevance of the network needs to be evaluated in reference to the disease under investigation. By using networks for non-brain tissues and other brain regions, all we wanted to show is that results change by tissue and that, when a tissue unrelated to Alzheimer’s disease is investigated, the selected gene(s) do not show biological relevance to the disease. In the case of *ZBBX* (<https://www.genecards.org/cgi-bin/carddisp.pl?gene=ZBBX>), this gene does not map to any known pathways and we could not find evidence in the scientific literature of links with Alzheimer’s disease. We added a clarifying note to this effect in the Discussion (page 22, lines 419-426).

========================================================================

**Reviewer #3:**

1. *The authors present NETPAGE, a method to investigate how rare variants spread in gene interaction networks. NETPAGE combines a network propagation approach with LASSO regression to find genes that are robustly high-scoring when changing a sparsity parameter. The authors apply NETPAGE to two Alzheimer's GWAS datasets, find high-scoring genes, and analyze the neighborhood of these genes. The paper is well written and clear, the results are interesting to read, and the authors do a good job of performing different complementary analyses on the NETPAGE results (e.g. survival analysis, gene set enrichment, RNA-seq overexpression).*

We thank the referee for their accurate summary and encouraging evaluation of our work. However, we would like to clarify one point: NETPAGE was applied in the context of Alzheimer’s disease, but not to GWAS datasets. GWAS datasets typically focus on common variants (i.e., with MAF > 1%), whereas our focus here was on **rare** **variants**. This is why we did not make use of the many, highly standardised SNP microarray datasets available, but we applied NETPAGE to whole-genome and whole-exome sequencing datasets. In addition, NETPAGE works on individual-level data rather than on summary statistics for prioritizing genes for follow-up analyses. Thus, NETPAGE’s objective is more closely related to SKAT-O and related tests than to existing tools using network propagation.

2. *However, I have two related concerns. First, NETPAGE is not a particularly novel method. There are many methods that do some type of network-based diffusion to find genes associated with a disease. The authors mention approaches such as CATAPULT, HotNet2, and NBS in the discussion but note that these are not appropriate to benchmark because they are fundamentally solving different problems. However, these approaches and others perform a similar type of analysis - for example, HotNet2 has been applied to complex diseases beyond cancer [Nakka 2016]. Further, [Lee 2011] models uncertainty of GWAS associations for network diffusion methods. Finally, [Lancour 2018] seems to have nearly the same goals as NETPAGE, using a network-diffusion approach to improve prediction of disease genes, and applied it on a large Alzheimer's GWAS dataset. These papers are the first of many that the authors need to either justify how their approach is substantially different or directly compare in the manuscript.*

Once again we thank the reviewer for pointing us to relevant literature in the context of which our work has to be justified. As briefly mentioned in the previous answer, the application of network propagation to rare variants and on individual level data is what makes this work substantially different from - and in many ways, complementary to - the other publications cited by the reviewer.

We agree that network propagation in itself is not a novel paradigm to boost detection of genetic associations with diseases; in fact, what [Lee 2011], [Nakka 2016] and [Lancour 2018] all have in common is the application to common variants (SNPs), where effect sizes are typically very small but sample size is not a limiting factor (thanks to the relatively contained costs of SNP genotyping) and therefore statistical power can be rescued. In this work, we wanted to investigate whether network propagation is a tool powerful enough to boost genetic associations in the case of rare variants, where low frequency and small sample sizes are important limiting factors for power. Additionally, [Lee 2011] and [Nakka 2016] developed their methods to be applied to association summary statistics: [Lee 2011] on gene-level statistics, [Nakka 2016] on SNP-level p-values. Similarly, [Lancour 2018] marks known associated AD genes in an interaction network and then applies diffusion. In contrast, **our method requires individual-level genotypes**. In summary, the papers highlighted by the referee perform first some association testing and then apply network propagation, whereas our method first applies network propagation and then performs association testing.

We added a paragraph in the discussion (page 22, lines 423-427) to clarify the differences between this branch of network propagation literature and our approach: “*There are also several recent studies where network propagation has been leveraged to boost power of genetic association testing with remarkable improvements [31–33]; however, these applications are substantially different from NETPAGE, in that the focus there was on common variation (GWAS datasets) while here we set ourselves the more challenging task of investigating rare variants in NGS experiments.*”

3. *Second, the authors do not adequately justify that their method is finding biological signal within the datasets. One way to demonstrate this is through a classification-type framework, which they explicitly avoid since their problem is not one of a classification task. However, in that case, the subsequent simulations are even more important for their justification of the method. The simulations mutate some combination and percentage of first and second neighbors for a target protein - this needs to consider how we expect SNVs to be distributed through the network.*

We would like to point out that we performed several follow-up analyses to show that the method is finding biological signal within the dataset: the analysis of added predictive value for the smoothed scores (pseudo-R2 statistic); the significant associations with risk for clinical conversion found in survival analysis; and the tissue-specific differential expression analysis all provide strong evidence for the biological plausibility of the associations found.

Additionally, it is widely recognised in the statistics community that strong statistical association between a variable and an outcome does not automatically imply that the variable is able to predict the outcome on a holdout sample with a sufficient level of accuracy [1]. Well-powered genome-wide association studies typically identify strong associations, but these results are never used for classification and their predictive performance is never evaluated; nonetheless GWAS are nowadays an established framework and have enabled many ground-breaking discoveries in the genetics of complex traits and diseases. Following a similar line of thought, we did not regard classification as an appropriate approach to answer the research question under investigation.

As for the simulated gene mutation burdens, these were designed to be consistent with the expected number of loss-of-function variants in the human genome and with their distribution through the network. Specifically, MacArthur et al. [2] found that a sample healthy subject of European ancestry carries 97 loss-of-function variants, of which 18 present in a homozygous state. In our work, in the scenario where both first and second neighbours of the target gene (CALML3) were mutated, the mutation burden spanned 2,000 subjects and 4,605 genes. With mutation frequencies of 0.5% in healthy controls and 1% in AD cases (the highest mutation frequencies allowed), we generated a distribution of rare deleterious variants that is displayed in the histogram below. In blue the distribution of the number of SNVs simulated in the network for healthy controls, in orange the same distribution for AD cases. In our simulations, a single healthy subject does not carry more than 35 SNVs (assuming one SNV per gene). Considering that the whole hippocampus network contains approximately three times the number of genes, the projection for the simulated genome-wide number of SNVs agrees very well with the estimate of 97 variants from MacArthur et al. Our simulated SNV burdens are therefore realistic and representative of the expected number and distribution of SNVs throughout the human genome and interactome.

[1] Shmueli, Galit. "To explain or to predict?." *Statistical science* 25.3 (2010): 289-310. <https://projecteuclid.org/euclid.ss/1294167961>

[2] MacArthur DG, Balasubramanian S, Frankish A, Huang N, Morris J, Walter K, et al. “A systematic survey of loss-of-function variants in human protein-coding genes”. *Science* (80- ). 2012;335: 823–828. doi:10.1126/science.1215040

4. *Further, the simulations are run on subnetworks with first and second neighbors only, which effectively removes the potential signal from more distantly related genes.*

We recognise this as a limitation of our simulation framework. The choice of subnetworks with first and second neighbours only was motivated by computational resources and speed, due to the high number of parameter combinations investigated. Additionally, the focus of our simulation framework was to ensure that the network propagation step was working as expected in our implementation and to map out some reasonable default parameters. We did not intend to investigate the path length that a signal is able to effectively travel in order to be detected in the core hub gene.

However, an analysis of network topology shows that in the hippocampus functional network the largest connected component has an average shortest path length of 3.33. This means that, on average, the shortest path between any two nodes is made of three steps. A subnetwork of radius 2, like the one in Figure 3C, has average shortest path length of 2.85. Given that virtually any gene in the network can be reached in three steps, we believe that a subnetwork of radius 2 is a good approximation of the global network topology and that the loss of “potential signal from more distantly related genes” is a negligible risk.

5. *When the authors run NETPAGE on the two AD datasets, Fig S3 shows that these results are not recovered when using the non-smoothed scores, using a randomized brain network, or using another tissue network. However, are the best-performing genes the ones that are reported for the AD datasets on the original brain network? [...] If the trends (e.g. relative order of important nodes) are consistent when randomizing the network or using non-smoothed scores, this could indicate that NETPAGE amplifies the signal but does not make new discoveries.*

We thank the reviewer for another very insightful comment. We looked at the ranks of genes according to their selection probability after smoothing through different networks, and at their pairwise correlations through Kendall’s tau. Below some examples:

| Tissue network used for propagation | Rank correlation with NETPAGE results using hippocampus network (Kendall’s tau) |
| --- | --- |
| None (“raw” case, Figure S3-A) | 0.22 |
| Randomised hippocampus network (Figure S3-B) | 0.35 |

It can be observed that the trends (e.g., relative order of important nodes) when randomizing the network or using non-smoothed scores show some consistency across networks, although this consistency is low. We see this as an attractive feature of the presented method, and as additional proof that NETPAGE is serving the purpose for which it was developed. Namely, to amplify the rare variant signal such that a gene that normally would not survive variable selection is now picked - so in a sense it would not be a new discovery, only a true signal that was originally too weak to be uncovered.

6. *Why use the 80% cutoff?*

This is recommended by the developers of both the stability selection framework [Meinshausen & Buhlmann] and of the R package stabs [Hofner & Hothorn] - in the package vignette they recommend using 75% selection probability as cutoff, we decided to adopt a higher cutoff to limit the rate of false discoveries and be more confident in robust gene associations.

7. *The application of NETPAGE to two different AD datasets reveals some intriguing overlaps related to PFAS and the neighborhood of PFAS genes. They compare to SKAT-O, but again it is unclear whether the NETPAGE results are biologically relevant so the comparison to SKAT-O is hard to interpret.*

We would like to point out again that we performed several follow-up analyses to show the biological relevance of the results: the analysis of added predictive value for the smoothed scores (pseudo-R2 statistic); the gene-set enrichment analysis; and the significant associations with risk for clinical conversion found in survival analysis; and the tissue-specific differential expression analysis all provide strong evidence for the biological plausibility of the associations found. Additionally, we found numerous studies documenting the link between *PFAS*, the glutamine-glutamate cycle, purine metabolism and its implications for Alzheimer’s disease. And finally, for other genes that the current literature does not characterise as directly relevant to Alzheimer’s disease (such as *CAMK2B*), we demonstrated also graphically (Figure 3C) that they are hubs for many known and validated Alzheimer’s genes. The comparison to SKAT-O in our opinion has a straightforward interpretation in the sense that, starting from the same set of genetic mutations and respective genes, our method is able to find meaningful associations while SKAT-O is not.

8. *They conduct a number of other experiments on the resultant genes and neighborhoods. First, the choice of focusing on neighborhoods is an intuitive one but not necessarily well-justified in terms of the results. Why not look at direct neighbors only?*

As the reviewer rightly pointed out in one of their previous comments about our simulation approach, network propagation enables signals to travel from distantly related genes and to converge into a network hub. The choice to look at gene-set enrichments for the set of first and second neighbours was motivated exactly by this assumption. It was also justified by the observation that many Alzheimer’s genes are indeed often at a distance of 2 links from the hub (Figure 3C in the main text). Additionally, including only first neighbours would have resulted in a relatively small gene set (41 genes), that is typically detrimental to the performance of GSEA.

9. *It is unclear what the outcome of the gene set enrichment analysis is: some of the significant gene sets are related to Alzheimer's, but the paper reports thousands of gene sets and it is unclear whether the AD-related gene sets would rise to the top of this list.*

We thank the reviewer for pointing out that the presentation of these results could be improved. We would like to clarify that, although we reported significant enrichments in 1,815 gene sets, the number of gene sets tested for enrichment was much higher (10,186). Therefore only a small fraction of the gene sets tested showed significant overlap with the set of first and second neighbours of PFAS (1,449 genes). When conducting GSEA, we were simply seeking to test the hypothesis of significant overlaps with AD gene sets, but there would be no reason to expect these overlaps to rise to the top of the list of significant sets. In fact, the five significant AD sets are widely distributed across this list (with the first ranked 41st and the last ranked 1,180th), but we do not see this as an issue. The intended positive outcome for this analysis was to show significant overlaps after FDR correction (and indeed it so happens for five out eight curated gene sets), not to prove that these overlaps are stronger than any other possible ones. What was more important for us was to prove was the robustness of these overlaps with AD sets through randomisation control (Supplementary Figure 11), which clearly showed that these enrichments could not have been achieved by chance only because of the size of the set under test.

10. *Since PFAS is such an integral part of the results, the role of this gene and resulting mutations deserves more discussion.*

The genes identified by stability selection exhibit connections to a number of intermediate phenotypes and biochemical processes relevant to AD. For instance, there are multiple, distinct lines of evidence linking *PFAS* (phosphoribosylformylglycinamidine synthase) to Alzheimer’s Disease. Common missense variation in *PFAS* has been linked to low-density lipoprotein (LDL) cholesterol measurement through GWAS [31,32], and elevated LDL cholesterol is reportedly a risk factor for AD, through an increased production of beta-amyloid protein [reference #1 below]. Additionally, Gene Ontology annotations for *PFAS* include the term ‘glutamine metabolic process’ (GO:0006541): the metabolism of glutamine and the glutamine-glutamate cycle takes place in the brain at the astrocyte level [33], and disruptions in this cycle leading to glutamate excess in the synaptic cleft trigger neuronal excitotoxicity, which has been implicated in AD [34]. But most importantly, *PFAS* is involved in the purine metabolism pathway (see for instance the KEGG pathway hsa00230), whose purpose in humans is to maintain an optimal level of the nucleotides in the tissues. Several studies have implicated perturbations in purine metabolism in the mechanisms of neurodegenerative disorders, including AD. For instance, Xiang et al [ref #4] found evidence for a role of the purine metabolic pathway through an integrative approach bringing together GWAS, KEGG pathways and gene expression; proteomic profiling in Kaddurah-Daouk et al [ref #3] showed that the purine pathway is directly related to CSF total tau; Ansoleaga et al [ref #5] showed stage- and region-dependent deregulation of purine metabolism in AD by analysing mRNA expression levels of purine metabolism genes in entorhinal cortex samples (the entorhinal cortex surrounds the hippocampus and is one of the earliest regions to be affected by tau accumulation in AD). Lastly, González-Domínguez et al [ref #6] showed metabolic alterations compatible with deregulation of the purine pathway mainly localised in the hippocampus of the APP/PS1 mouse model of AD. Hence it seems plausible that rare deleterious variation in *PFAS* or its functional neighbourhood should increase susceptibility to AD through its impact on cholesterol and glutamate levels as well as the synthesis and metabolism of purines.

We integrated the Discussion section in the main text with the paragraph above.

1. Puglielli, L., Tanzi, R. & Kovacs, D. Alzheimer's disease: the cholesterol connection. *Nat Neurosci* 6, 345–351 (2003). https://doi.org/10.1038/nn0403-345
2. KEGG PATHWAY: hsa00230 (<https://www.genome.jp/dbget-bin/www_bget?pathway:hsa00230>)
3. Kaddurah-Daouk, R., Zhu, H., Sharma, S. *et al.* Alterations in metabolic pathways and networks in Alzheimer’s disease. *Transl Psychiatry* 3, e244 (2013). https://doi.org/10.1038/tp.2013.18
4. Xiang, Z., Xu, M., Liao, M. *et al.* Integrating Genome-Wide Association Study and Brain Expression Data Highlights Cell Adhesion Molecules and Purine Metabolism in Alzheimer’s Disease. *Mol Neurobiol* 52, 514–521 (2015). https://doi.org/10.1007/s12035-014-8884-5
5. Ansoleaga, Belén, et al. "Deregulation of purine metabolism in Alzheimer's disease." *Neurobiology of aging* 36.1 (2015): 68-80.
6. Gonzalez-Dominguez, Raul, et al. "Region-specific metabolic alterations in the brain of the APP/PS1 transgenic mice of Alzheimer's disease." *Biochimica et Biophysica Acta (BBA)-Molecular Basis of Disease* 1842.12 (2014): 2395-2402.

*11. There is a substantial section of the data availability in the Supplementary Methods. However, it is unclear what "Some restrictions will apply" means in the submission questions.*

The statement "Some restrictions will apply" refers to the fact that the ADNI WGS and ADSP WES datasets are available for research purposes conditional to a formal application and the availability of appropriate and secure data storage capabilities. The remainder of the datasets used in this work is publicly available without restrictions.

*Minor Comments:*

*- Figure 2 should use "cases", "controls", "diffusion length" and "quantile normalization" as labels.*

We modified Figure 2 to display the correct labels.

*- In the Results under "Medium sample:ADSP", what is the "PFAS interactome"? I understand that PFAS is the predicted gene from the ADNI samples, but how this makes an interactome needs more clarification.*

The use of the word “interactome” in this context was incorrect. We have replaced it with “neighbourhood”, as the reference here would be to the interaction partners (or network neighbours) of *PFAS*.

*- Is there a reason why the authors considered binary variants rather than some p-value or risk score for SNVs?*

Yes, the reason being that we wanted to apply network propagation to boost the information content of genes BEFORE performing association testing, not after (like it is done in other published works). Please refer to the answers to the reviewer’s first two major comments for further details.
